# Association of Body Mass Index With Clinical Outcomes in Patients With Cystic Fibrosis: A Systematic Review and Meta-analysis

**DOI:** 10.1001/jamanetworkopen.2022.0740

**Published:** 2022-03-07

**Authors:** Rita Nagy, Noémi Gede, Klementina Ocskay, Bernadett-Miriam Dobai, Alan Abada, Zsófia Vereczkei, Piroska Pázmány, Dorottya Kató, Péter Hegyi, Andrea Párniczky

**Affiliations:** 1 Institute for Translational Medicine, Szentágothai Research Centre, Medical School, University of Pécs, Pécs, Hungary; 2 Centre for Translational Medicine, Semmelweis University, Budapest, Hungary; 3 Heim Pál National Pediatric Institute, Budapest, Hungary; 4 George Emil Palade University of Medicine, Pharmacy, Science and Technology of Targu Mures, Romania; 5 Department of Anaesthesiology and Intensive Therapy, Medical School, University of Pécs, H-7624 Pécs, Hungary; 6 Doctoral School of Clinical Medicine, University of Szeged, Szeged, Hungary; 7 Division of Pancreatic Diseases, Heart and Vascular Center, Semmelweis University, Budapest, Hungary

## Abstract

**Importance:**

The prevalence of overweight (body mass index [BMI] = 25-29.9 [calculated as weight in kilograms divided by height in meters squared]) and obesity (BMI ≥30) is increasing among patients with cystic fibrosis (CF). However, it is unclear whether there is a benefit associated with increasing weight compared with the reference range (ie, normal) in CF.

**Objective:**

To evaluate the association of altered BMI or body composition and clinical outcomes in patients with CF.

**Data Sources:**

For this systematic review and meta-analysis, the literature search was conducted November 2, 2020, of 3 databases: MEDLINE (via PubMed), Embase, and Cochrane Central Register of Controlled Trials.

**Study Selection:**

Patients older than 2 years diagnosed with CF with altered body composition or BMI were compared with patients having the measured parameters within the reference ranges. Records were selected by title, abstract, and full text; disagreements were resolved by consensus. Cohort studies and conference abstracts were eligible; articles with no original data and case reports were excluded.

**Data Extraction and Synthesis:**

Two authors independently extracted data, which were validated by a third author. Studies containing insufficient poolable numerical data were included in the qualitative analysis. A random-effects model was applied in all analyses.

**Main Outcomes and Measures:**

Pulmonary function, exocrine pancreatic insufficiency (PI), and CF-related diabetes (CFRD) were investigated as primary outcomes. Odds ratios (ORs) or weighted mean differences (WMDs) with 95% CIs were calculated. The hypothesis was formulated before data collection.

**Results:**

Of 10 524 records identified, 61 met the selection criteria and were included in the qualitative analysis. Of these, 17 studies were included in the quantitative synthesis. Altogether, 9114 patients were included in the systematic review and meta-analysis. Overweight (WMD, –8.36%; 95% CI, –12.74% to –3.97%) and obesity (WMD, –12.06%; 95% CI, –23.91% to –0.22%) were associated with higher forced expiratory volume in the first second of expiration compared with normal weight. The odds for CFRD and PI were more likely in patients of normal weight (OR, 1.49; 95% CI, 1.10 to 2.00) than in those who were overweight (OR, 4.40; 95% CI, 3.00 to 6.45). High heterogeneity was shown in the analysis of pulmonary function (*I*^2^ = 46.7%-85.9%).

**Conclusions and Relevance:**

The findings of this systematic review and meta-analysis suggest that the currently recommended target BMI in patients with CF should be reconsidered. Studies with long-term follow-up are necessary to assess the possible adverse effects of higher BMI or higher fat mass in patients with CF.

## Key Points

**Question:**

Is higher-than-normal body mass index (BMI) associated with better clinical outcomes in patients with cystic fibrosis?

**Findings:**

In this systematic review and meta-analysis of studies including 9114 patients with cystic fibrosis, BMI indicating overweight and obesity were associated with better pulmonary function and lower chance for exocrine pancreatic insufficiency and cystic fibrosis–related diabetes compared with normal BMI.

**Meaning:**

The findings of this study suggest that guidelines should be updated to recommend a higher target BMI in patients with cystic fibrosis.

## Teaser

This systematic review and meta-analysis compares the clinical outcomes of patients with cystic fibrosis by body mass index.

## Introduction

Cystic fibrosis (CF) is a common, often lethal inherited disorder caused by recessive genetic variants in the CF transmembrane conductance regulator (*CFTR*) gene affecting 1 in 3300 neonates of White race.^1^ The variants result in diminished function of chloride, sodium, and bicarbonate ion transport, leading to thick mucus production that causes functional derangement of multiple organs (eg, lungs, gastrointestinal system, and reproductive system).^2^ Consequential frequent airway infections, chronic inflammation, exocrine pancreatic insufficiency (PI), and complications, such as CF-related diabetes (CFRD) and CF-related liver disease, result in general unease and poor quality of life.^3^

Among patients with CF, malnutrition is commonly seen, mainly caused by the combination of the following mechanisms: (1) nutrient malabsorption and fecal energy loss due to PI, (2) increased energy expenditure predominantly related to chronic inflammation and breathing efforts,^4,5,6^ and (3) loss of appetite caused by inflammation-related anorexia.^7^ Malnourishment may accelerate the progression of the disease; nevertheless, underweight (body mass index [BMI]<15th percentile, with BMI calculated as weight in kilograms divided by height in meters squared) is known to be associated with worse pulmonary function and increased morbidity and mortality in patients with CF.^8^

Monitoring the nutritional status and growth of patients, as well as the prevention and treatment of malnutrition, are demanding components of CF care. Currently, BMI is the generally accepted indicator for monitoring the nutritional status of patients with CF. In children older than 2 years, the target BMI is at least the 50th percentile; in adults, the target BMI is greater than or equal to 22 for women and greater than or equal to 23 for men.^9^ However, BMI does not distinguish between the major components of the body, namely, fat mass (FM), fat-free mass (FFM), total body water, bone mineral density, and bone mineral content. There is a growing body of evidence highlighting the importance of nutritional status in the diagnostic and therapeutic management of patients with CF,^10,11,12^ but there is a lack of comprehensive review on patients with a BMI above the target.

The European Society for Clinical Nutrition and Metabolism; the European Society for Paediatric Gastroenterology, Hepatology, and Nutrition; and the European Cystic Fibrosis Society adult and pediatric dietary guideline focuses on nutritional failure with no recommendation on the management of individuals who are overweight or obese.^9^ However, an analysis of BMI changes found that the prevalence of overweight and obesity in adults with CF is 31.4% and has more than doubled over the past 2 decades in patients with CF.^13^ However, long-term adherence to the currently recommended high-fat and high-carbohydrate diet in CF also might have controversial effects on body composition and even on some clinical outcomes. It is unclear whether there is an advantage of increasing weight over the normal range in CF. For instance, mortality in pneumonia has been reported to be lower in individuals without CF who are obese, known as the obesity survival paradox.^14^ To fulfill the knowledge gap, we aimed to evaluate the differences in clinically significant outcomes, such as lung function, PI, and CFRD, in patients with CF having altered BMI and/or body composition by conducting a systematic review and meta-analysis of the literature.

## Methods

The review protocol for this systematic review and meta-analysis was prospectively registered with PROSPERO. The only deviation from our protocol was the addition of *Pseudomonas aeruginosa* colonization incidence. Findings are reported in accordance with the Preferred Reporting Items for Systematic Reviews and Meta-analyses (PRISMA) reporting guideline.^15^

### Study Selection

The literature search was conducted November 2, 2020, in MEDLINE (via PubMed), Embase, and Cochrane Central Register of Controlled Trials. Key search terms included *cystic fibrosis*, *body fat*, *body mass*, and *body weight* without any restrictions. Further details regarding the strategy of the literature search and selection can be found in the eMethods in the Supplement.

Two of us (R.N. and P.P.) independently conducted the selection in duplicate using reference management software (Endnote X9 software; Clarivate Analytics; 2019). Removal of duplicates was performed automatically and after that manually. The records were selected by title, abstract, and full text based on a previously determined set of rules. Any disagreements were resolved by consensus between the 2 reviewers. After each step of the selection process, the rate of agreement was determined and documented by calculating the Cohen κ coefficient. Values may indicate slight (0-0.20), fair (0.21-0.40), moderate (0.41-0.60), substantial (0.61-0.80), and almost perfect agreement (0.81-1.00).^16^ References of each included study were checked, and records that were considered to be eligible were added to the pool.

### Eligibility Criteria

Cohort studies, case series, and clinical trial or conference abstracts were eligible; case reports and articles with no original data were excluded from our systematic review. The research question was formulated using the Population, Exposure, Comparator, and Outcomes framework.^17^

Patients older than 2 years diagnosed with CF regardless of sex, transplant status, *CFTR* modulator therapy, or comorbidities with altered body composition (BMI, FFM, and FM values out of the reference ranges, eg, underweight, overweight, and obese) were compared with patients with the measured parameters within the reference ranges. Articles reporting coefficients regarding the association between BMI or body composition and clinical outcomes were also eligible.

The nutritional categories were accepted based on study definition; however, we intended to strictly follow the thresholds recommended by the World Health Organization^18^: underweight (BMI <18.5), normal weight (BMI = 18.5-24.9), overweight (BMI ≥25), and obese (BMI ≥30) when it was possible to analyze separately. We also compared the underweight group (BMI <20) with the nonunderweight group (≥20) and performed subgroup analyses based on the age of the participants (adults, children, and mixed population).

Primary outcomes included pulmonary function (expressed by forced expiratory volume in the first second of expiration [FEV_1_%]), PI, and CFRD. Diagnosis of PI and CFRD was determined according to the definitions used in the included studies. As secondary outcomes, we investigated parameters associated with metabolic status, including fasting glucose, fasting insulin, hemoglobin A_1c_ (HbA_1c_), cholesterol, and triglyceride levels, and *P aeruginosa* colonization as an additional outcome.

### Data Extraction

Two of us (R.N. and D.K) independently extracted data into a standardized data collection sheet (Excel 2019; Microsoft Corp), and data extraction was validated by another one of us (B-M.D.). Data collected included sex distribution, age distribution, genotype, patient numbers, and mean or median values of outcomes of interest. Correlation coefficients were also extracted regarding the association of BMI or body composition and clinical outcomes. Further details regarding data extraction can be found in the eMethods in the Supplement. Most of the eligible studies were cross-sectional. For longitudinal studies, we collected only baseline data. For overlapping populations, the study working with the most patients was chosen for each outcome.

### Risk of Bias Assessment

Based on the recommendations of the Cochrane Prognosis Methods Group, the Quality in Prognostic Studies (QUIPS) tool was applied by 2 of us (R.N. and P.P.) to assess the risk of bias in the included studies for each outcome separately.^19^ Any disagreement was resolved based on consensus.

### Statistical Analysis

A random-effects model was applied in all analyses using the DerSimonian-Laird estimation.^20^ Pooled odds ratios (ORs) with corresponding 95% CIs were calculated for dichotomous outcomes. Pooled mean difference was calculated for continuous outcomes (weighted mean difference [WMD]). Results of the meta-analyses are displayed in forest plots. Statistical heterogeneity was analyzed using the *I*^2^ and χ^2^ tests to gain probability values; *P* < .10 was defined to indicate significant heterogeneity. *I*^2^ values representing moderate (30%-60%), substantial (50%-90%), and considerable (75%-100%) heterogeneity were based on the Cochrane Collaboration recommendations.^21^ Sensitivity analyses were also carried out omitting 1 study and calculating the summary OR or WMD with the 95% CI to investigate whether there was an association between a single study and the final estimation. To check for publication bias, a visual inspection of funnel plots was performed with Egger tests.^22^ Statistical analyses were carried out using Stata, version 16 SE (StataCorp LLC). For continuous variables, *P* values were calculated using 2-tailed unpaired analysis. Results were considered significant at *P* < .05.

## Results

### Study Selection

**Figure 1.**
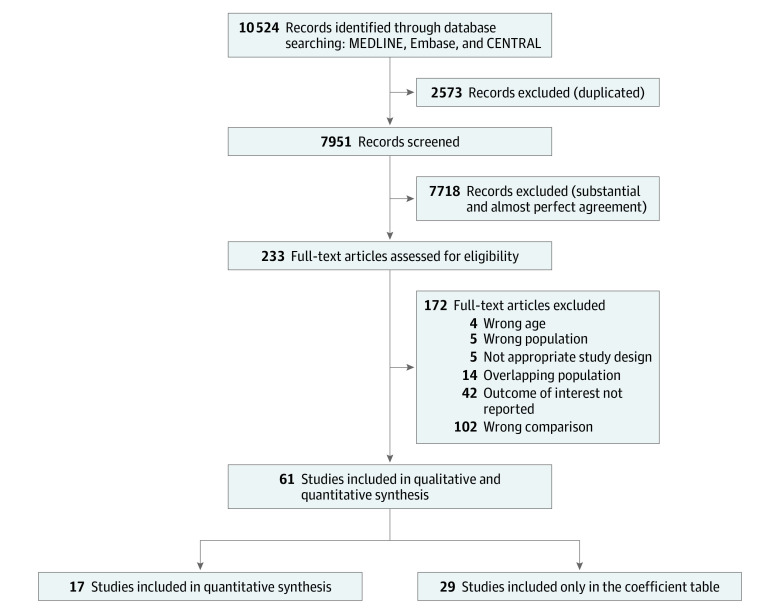
Preferred Reporting Items for Systematic Reviews and Meta-analyses Flowchart CENTRAL indicates Cochrane Central Register of Controlled Trials.

The systematic literature search yielded 10 524 records. After removal of duplicates, 7951 records were screened; of these, 61 records were included in the qualitative analysis and 16 full-text articles and 1 conference abstract were included in the quantitative analysis. Of the 61 studies, 33 contained correlational coefficients from which 29 did not report outcomes of interest according to BMI categories. These records and coefficients are included in the eTable in the Supplement. The selection process is shown in Figure 1.

### Study Characteristics

Altogether, 9114 patients were included in the systematic review and meta-analysis. Of 9114 patients, 5301 were included based on BMI categories, and studies that reported coefficients resulted in 3813 involved patients. Five studies investigated only children (<18 years), 13 studies included only adults, and 14 studies examined a mixed patient population. The estimated proportion of children (mixed population studies did not give the number of children) is 30%. The mean (SD) values of BMI in the analyzed groups ranged from 18.5 (1.7) to 34.8 (5.7). The major characteristics of the included studies are reported in the Table.

**Table.** Characteristics of Included Studies

Source	Characteristic					Setting		
	Patients, No.	Age group	Age, mean (SD), y	BMI	Pancreatic insufficiency, No. (%)	Genotype (DF508)	Growth parameter	Outcomes
Altman et al,^23^ 2017 (abstract)	224	Adults	32.4 (10.6)	NA	NA	NA	BMI	CFRD, *P aeruginosa* colonization
Alvarez et al,^24^ 2016	32	Mixed	26.1 (8.9)	22.1 (2.9)^a^	31 (96.9)	56.3% Homozygote; 28.1% heterozygote	BMI, percent body fat, FFM	FEV_1_%, CFRD, FG
Barni et al,^25^ 2017^b^	73	Mixed	25.6 (7.3)	21.0 (3.0)^a^	66 (90)	17.8% Homozygote; 45.2% heterozygote	BMI	FEV_1_%, PI, CFRD, *P aeruginosa* colonization
Bodnar et al,^26^ 2014^b^	59	Mixed	14.0 (4.8)	20.4 (2.4)^a^	NA	50.8% Homozygote	BMI, BMI percentile	FEV_1_%, *P aeruginosa* colonization
Bonhoure et al,^27^ 2020^b^	290	Adults	25.5 (7.9)	21.7 (2.9)^a^	232 (79.9)	49.0% Homozygote; 41.3% heterozygote	BMI	FEV1%, PI, CFRD, FG, HbA_1c_%, TC, TG, HDL-C, LDL-C, FI
Bouma et al,^28^ 2020	201	Mixed	13.3 (4.6)	19.9 (3.7)^a^	NA	51.2% Homozygote; 36.3% heterozygote	BMI, BMI *z* score	FEV_1_%
Brennan et al,^29^ 2010 (abstract)	348	Adults	No data	No data	NA	NA	BMI	FEV_1_%
Cano Megias et al,^30^ 2015^b^	61	Mixed	26.8 (9.5)	20.3 (3.3)^a^	NA	24.6% Homozygote; 78.6% heterozygote	BMI, *z* score	FEV_1_%
Charatsi et al,^31^ 2016	71	Mixed	12 (2.7)	18.2 (3.4)^c^	36 (90)	49.3% Homozygote; 39.4% Heterozygote	FFM *z* score	FEV_1_%, PI, *P aeruginosa* colonization
Da Silva Garrote et al,^32^ 2016 (abstract)	34	Children	10.2 (5.3)	No data	NA	NA	BMI percentile	*P aeruginosa* colonization
Dray et al,^33^ 2005^b^	163	Adults	28.8 (8.4)	19.1 (2.8)^a^	137 (84)	42.3% Homozygote; 38.6% heterozygote	BMI	PI, CFRD, *P aeruginosa* colonization
Dudina et al,^34^ 2017 (abstract)	435	Adults	No data	No data	NA	NA	BMI	FEV_1_%
Engelen et al,^35^ 2012^b^	77	Mixed	14.8 (2.9)	40.77 (26.4)^d^	75 (97)	63.6% Homozygote; 25.9% heterozygote	BMI, BMI percentile, FFM, *z* score	FEV_1_%
Gozdzik et al,^36^ 2008^b^	39	Adults	23.9 (3.7)	19.5 (2.9)^a^	NA	NA	BMI	FEV_1_%
Hanna and Weiner,^37^ 2015^b^	226	Children	10.6 (4.9)	18.5 (4.2)^a^	181 (80)	NA	BMI percentile	FEV_1_%, PI
Harindhanavudhi et al,^38^ 2020^b^	484	Adults	35.2 (11.6)	23.9 (4.4)^a^	417 (85)	46.9% Homozygote	BMI	FEV_1_%, PI, CFRD, HbA_1c_%, HDL-C, LDL-C
Hollander et al,^39^ 2018 (abstract)	224	Adults	No data	No data	NA	NA	BMI	FEV_1_%
Ionescu et al,^40^ 2003	56	Adults	23 (5.2)	20.9 (1.6)^a^	29 (100)	NA	FFM	FEV_1_%
González Jiménez et al,^41^ 2012^b^	109	Children	12.3 (8.8)	21.6 (3.9)^a^	371 (82)	41.3% Homozygote; 46.7% heterozygote	BMI percentile	FG, HbA_1c_%, TC, TG, FI
González Jiménez et al,^42^ 2017^b^	451	Mixed	12.7 (3.2)	−0.3 (0.8)^e^	93 (85)	33.0% Homozygote; 49.8% heterozygote	BMI, *z* score	FEV_1_%
Kines et al,^43^ 2012 (abstract)	114	Mixed	No data	No data	NA	NA	BMI	FEV_1_%
Kotsifas et al,^44^ 2016^b^ (abstract)	44	Adults	No data	No data	NA	NA	BMI	FEV_1_%
Maksimycheva et al,^45^ 2018 (abstract)	51	Children	No data	No data	NA	NA	BMI	FEV_1_%, *P aeruginosa* colonization
Ochota et al,^46^ 2019 (abstract)	226	Adults	No data	23.5 (6.0)^a^	NA	NA	BMI	FEV_1_%
Panagopoulou et al,^47^ 2008^b^	43	Mixed	20.1 (8.5)	19.4 (2.6)^a^	No data	NA	BMI, FFM	FI
Panagopoulou et al,^48^ 2014	68	Mixed	19.81(9.0)	19.8 (2.7)^a^	56 (82)	20.6% Homozygote; 48.5% heterozygote	BMI, BMI percentile	FEV_1_%, PI, CFRD, FG, TC, TG, *P aeruginosa* colonization
Papalexopoulou et al,^49^ 2018	29	Mixed	15 (1.8)	−0.1 (−2.7–1.2)^e^	28 (96.5)	58.6% Homozygote; 31.0% heterozygote	BMI *z* scores, BMI percentile, FFM index *z* score	FEV_1_%
Proud et al,^50^ 2012 (abstract)	117	Adults	28 (9)	No data	NA	NA	BMI, FFM index	FEV_1_%, PI
Ramírez et al,^51^ 2015^b^	173	Mixed	11.43 (2.3)	No data	145 (83.77)	50.3% Homozygote; 36.9% heterozygote	BMI percentile	FEV_1_%, PI, *P aeruginosa* colonization
Stephenson et al,^52^ 2013^b^	651	Adults	33.8 (11.4)	22.3 (4.1)^a^	488 (75)	39.3% Homozygote; 38.2% heterozygote	BMI	FEV_1_%, PI, CFRD, TC, TG, *P aeruginosa* colonization
Umławska et al,^53^ 2014^b^	89	Children	12.3 (3.5)	–0.8 (0.8)^e^	80 (90)	51.7% Homozygote; 33.7% heterozygote	BMI percentile	FEV_1_%, PI
Ziegler et al,^54^ 2008	39	Mixed	23.7 (6.4)	20.3 (2.2)^a^	NA	NA	BMI, BMI percentile	FEV_1_%

Abbreviations: BMI, body mass index; CFRD, cystic fibrosis–related diabetes; FEV_1_%, forced expiratory volume in the first second of expiration; FFM, fat-free mass; FG, fasting glucose; FI, fasting insulin; HbA_1c_, hemoglobin A_1c_; HDL-C, high-density lipoprotein cholesterol; LDL-C, low-density lipoprotein cholesterol; NA, not assessed; *P aeruginosa*, *Pseudomonas aeruginosa*; PI, exocrine pancreatic insufficiency; TC, total cholesterol; TG, triglycerides.

^a^ Value expressed as mean (SD).

^b^ Included in the quantitative synthesis.

^c^ Value expressed as median (IQR).

^d^ Value expressed as mean (SD) percentile.

^e^ Value expressed as *z* score.

### Primary Outcomes

#### Forced Expiratory Volume in the First 1 Second of Expiration

**Figure 2.**
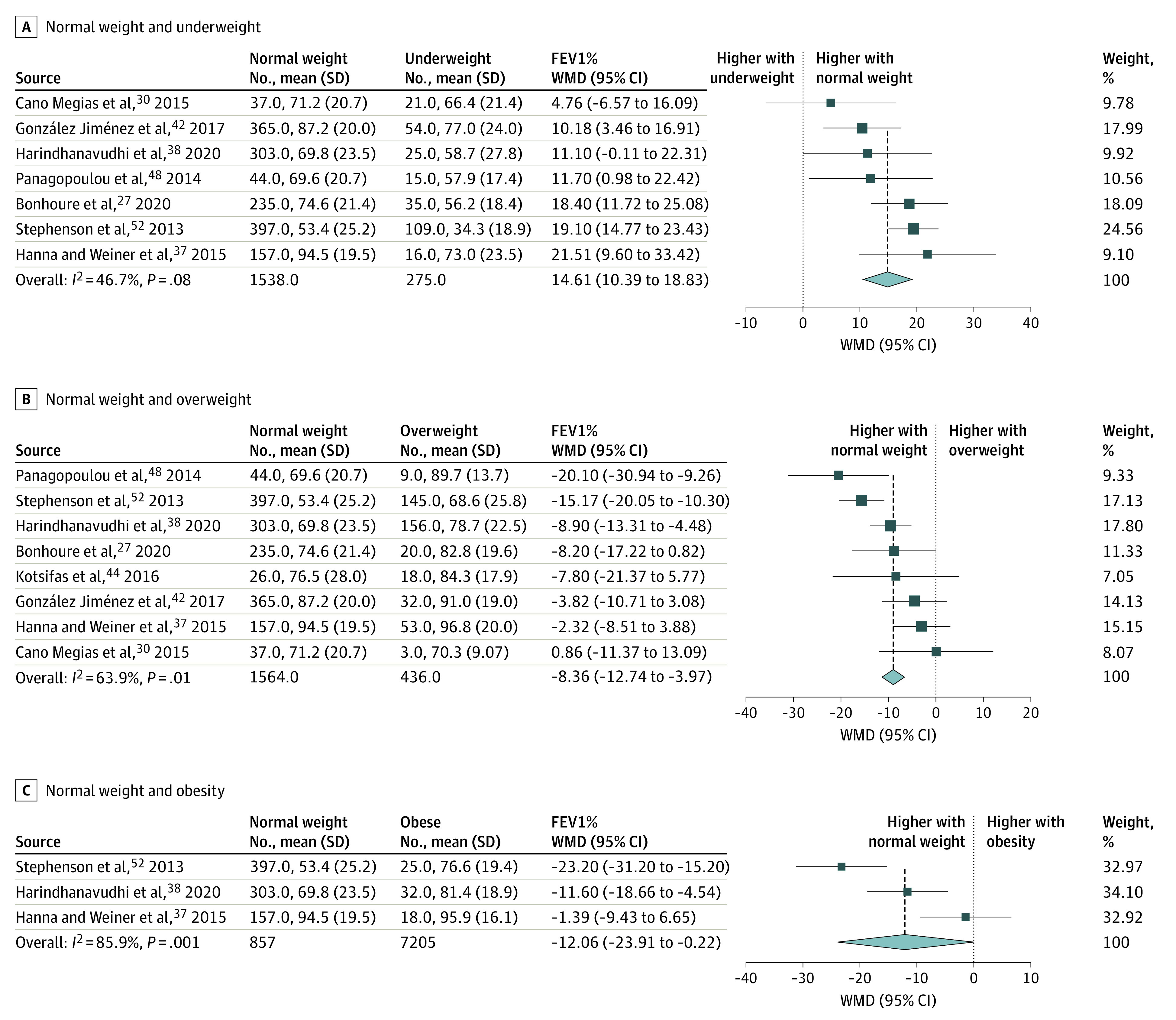
Pulmonary Function in Different Body Mass Index (BMI) Categories of Patients With Cystic Fibrosis Comparison of patients with normal weight vs underweight (moderate heterogeneity detected) (A), normal weight vs overweight (substantial heterogeneity detected) (B), and normal weight vs obesity (considerable heterogeneity detected) (C). FEV_1_% indicates forced expiratory volume in the first second of expiration; WMD, weighted mean difference. The size of squares is proportional to the weight of each study. Horizontal lines indicate the 95% CI of each study; diamond, the pooled estimate with 95% CI.

Most studies (54 of 61) reported FEV_1_% as an indicator of pulmonary function. A total of 13 studies were included in the quantitative synthesis. Based on our results, patients whose weight was considered normal had significantly higher FEV_1_% values compared with those who were underweight (WMD, 14.61%; 95% CI, 10.39%-18.83%). Compared with patients whose BMI was considered normal, better pulmonary function was noted in patients who were overweight (82.96% vs 74.60%; WMD, –8.36%; 95% CI, –12.74% to –3.97%) or obese (84.63% vs 72.57%; WMD, –12.06%; 95% CI, –23.91% to –0.22%) (Figure 2). High heterogeneity was shown in the analysis of pulmonary function (*I*^2^ = 46.7%-85.9%). In the comparison of patients who were underweight vs not underweight, we found significantly lower FEV_1_% in children, adults, and mixed patient populations who were underweight (overall WMD, –19.12%; 95% CI, –23.53% to –14.71%) (eFigure 1 in the Supplement).

In addition to the 13 records in the quantitative synthesis, 15 studies, including conference abstracts, were added to the qualitative synthesis. The reasons for exclusion of these 15 studies from the quantitative synthesis were either different BMI categorization from the World Health Organization recommendation or insufficient data reporting. Among these 15 studies, 9 studies showed increased FEV_1_% values to be associated with higher BMI values in patients with normal BMI or overweight compared with those who were underweight. Two studies did not report significant differences in pulmonary function when comparing different BMI categories.^49,54^ Five studies investigated the connection between pulmonary function and FFM, and all of the studies reported an association between FFM and pulmonary function. Most (39 of 42 [92.9%]) of the extracted correlation coefficients indicated significant correlation between BMI or body composition parameters and FEV_1_%. Considered one of the main possible confounders, use of modulator therapy was rarely reported (2 of 54 [3.7%]); therefore, we were not able to perform further analysis of these participants.

#### Exocrine Pancreatic Insufficiency

**Figure 3.**
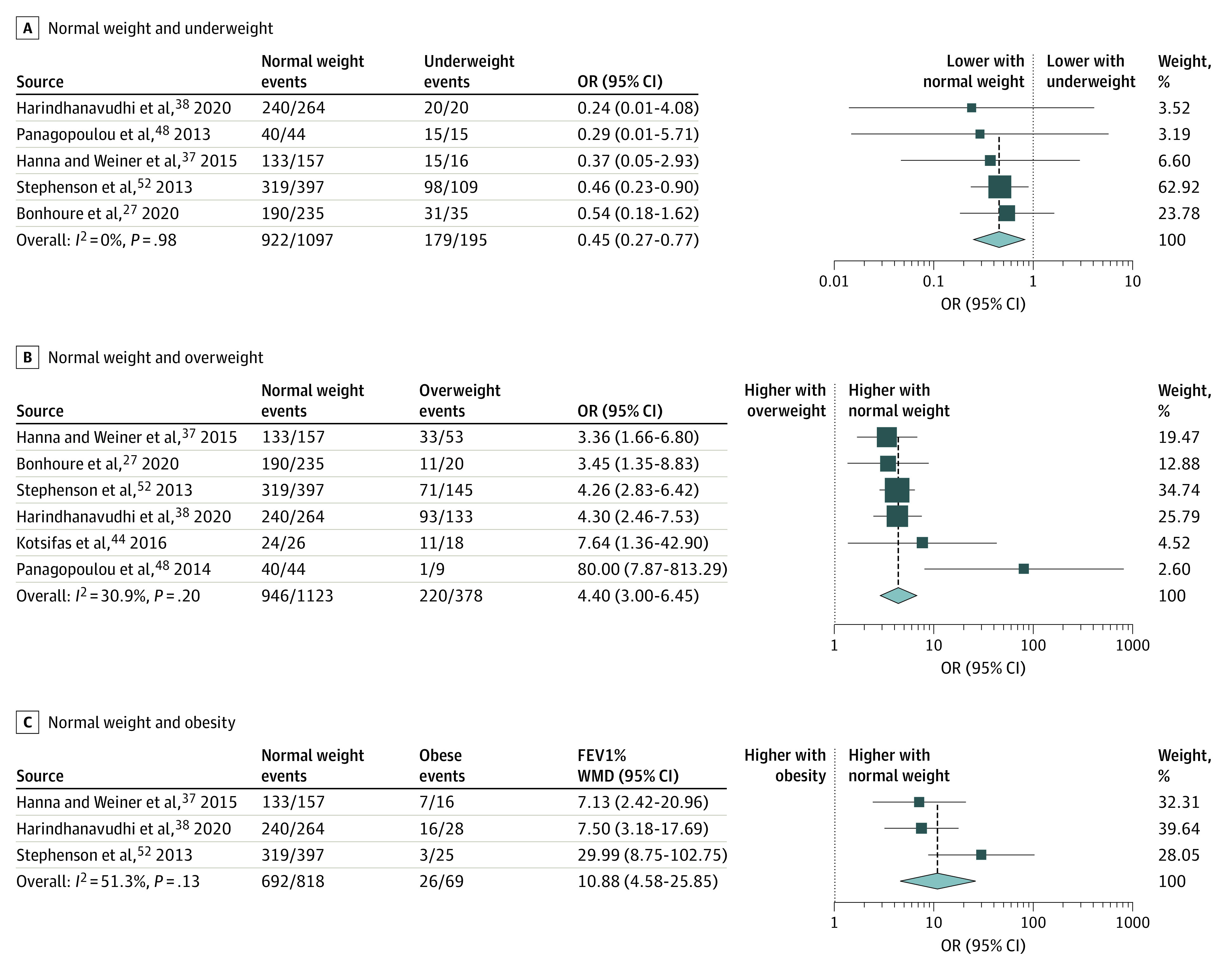
Odds of Exocrine Pancreatic Insufficiency in Different Body Mass Index Categories Comparison of patients with normal weight vs underweight (A), normal weight vs overweight (B), and normal weight vs obesity (C). OR indicates odds ratio. The size of squares is proportional to the weight of each study. Horizontal lines indicate the 95% CI of each study; diamond, the pooled estimate with 95% CI.

Our results showed that normal BMI is associated with a lower odds for PI compared with underweight (OR, 0.45; 95% CI, 0.27-0.77) and a higher likelihood of PI compared with overweight (OR, 4.40; 95% CI, 3.00-6.45) and obesity (OR, 10.88; 95% Cl, 4.58-25.85) (Figure 3). Adults who were underweight had significantly higher odds for PI (OR, 3.16; 95% CI, 1.97-5.06) compared with those of normal weight (overall OR, 2.54; 95% CI, 1.53-4.23) (eFigure 2 in the Supplement). The studies of Ramírez et al^51^ reported a nonsignificant result and were not included in our quantitative synthesis owing to inadequate data reporting.

#### CF-Related Diabetes

**Figure 4.**
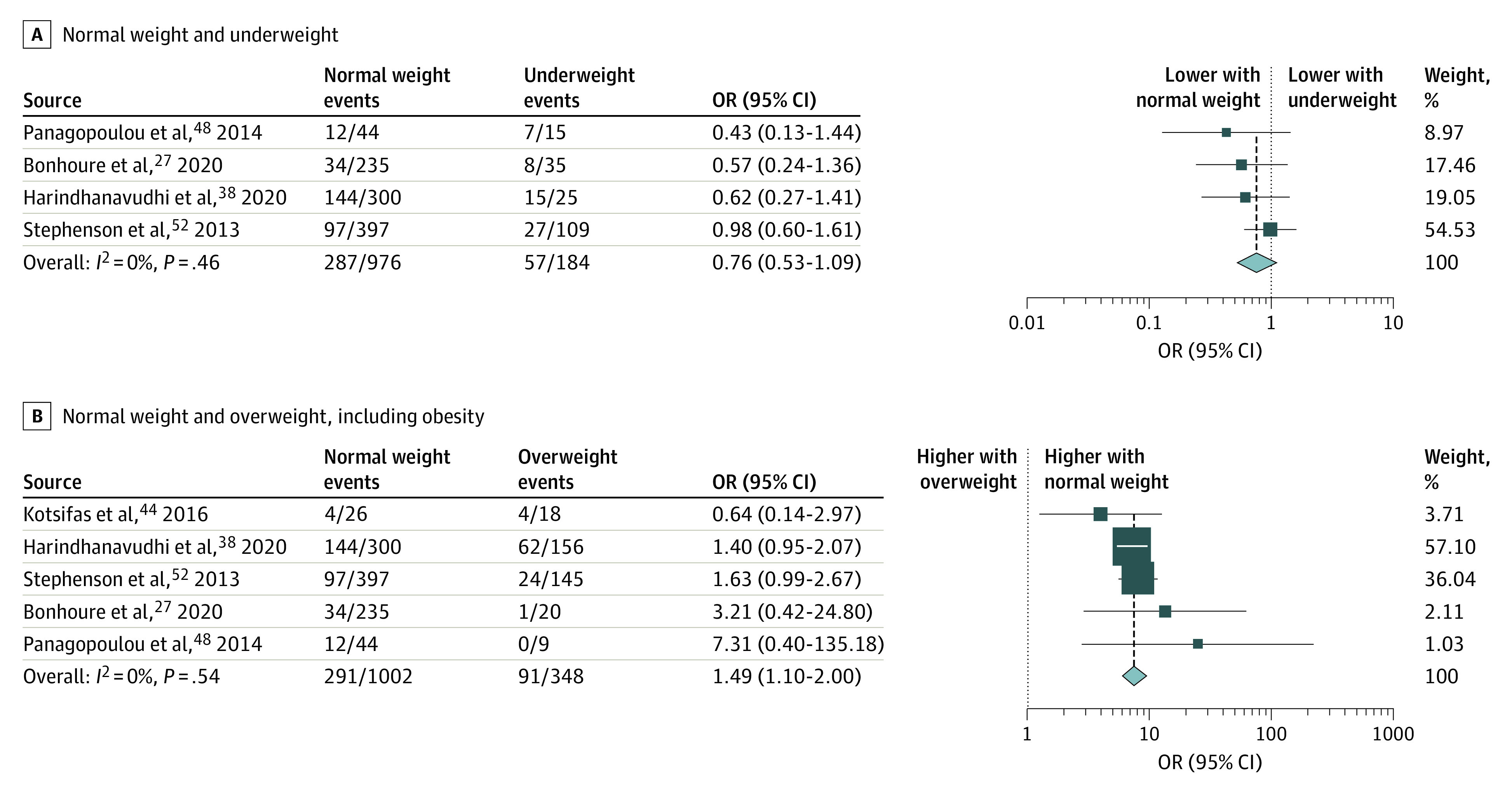
Odds for Cystic Fibrosis–Related Diabetes in Different Body Mass Index Categories Comparison of patients with normal weight vs underweight (A) and normal weight vs overweight and obesity. OR indicates odds ratio. The size of squares is proportional to the weight of each study. Horizontal lines indicate the 95% CI of each study; diamond, the pooled estimate with 95% CI.

Our results suggest that CFRD is more common in patients who are underweight compared with those who are of normal weight (31% vs 29.4%; OR, 0.76; 95% CI, 0.53-1.09). In addition, normal BMI is associated with higher odds for CFRD compared with overweight (OR, 1.49; 95% CI, 1.10-2.00) (Figure 4). Based on the subgroup analysis, the overall comparison showed significantly higher odds of CFRD in patients with lower BMI vs those with normal BMI (OR, 1.43; 95% CI, 1.04-1.9) (eFigure 3 in the Supplement). The studies of Altman et al^23^ and Alvarez and Stecenko^24^ were not included in our quantitative synthesis owing to inadequate comparison categories; however, none of the studies reported significant differences between BMI groups regarding the CFRD outcome.^24^

### Secondary Outcomes

Glucose metabolic status indicators, such as fasting glucose, fasting insulin, and HbA_1c_ levels did not significantly differ between BMI categories (eFigures 4-6 in the Supplement). However, in accordance with our hypothesis, compared with patients having normal weight, those who were overweight or obese had significantly higher total cholesterol levels (0.11 vs 0.09 mg/dL; WMD, –0.02 0.41 mg/dL; 95% CI, –0.03 to 0.01) and triglyceride levels (0.03 vs 0.02 mg/dL WMD, –0.005; 95% CI, –0.009 to 0.0005 [to convert to millimoles per liter, multiply by 0.0113]) (eFigure 7 and eFigure 8 in the Supplement). In the comparison of patients with normal weight vs underweight, both cholesterol levels (WMD, 0.008 mg/dL; 95% CI, 0.004 to 0.013) and triglyceride levels (WMD, 0.003 mg/dL; 95% CI, 0.001 to 0.006) were significantly higher in the normal weight group (eFigures 7-8 in the Supplement). Bonhoure et al,^27^ Harindhanavudhi et al,^38^ and Panagopoulou et al^48^ reported high-density and low-density lipoprotein cholesterol values; all of the studies showed significantly higher low-density lipoprotein cholesterol levels and not significantly lower high-density lipoprotein cholesterol levels in patients who were overweight. However, these results could not be included in the quantitative synthesis owing to insufficient data reporting by Harindhanavudhi et al.^38^

### Additional Outcomes and Analysis

The ratio of patients with *P aeruginosa* colonization at the time of assessment was reported in 11 studies; of these, 6 studies were included in our quantitative synthesis. The results showed that patients who were underweight were significantly more likely to have *P aeruginosa* colonization compared with those who were not underweight (OR, 1.86; 95% CI, 1.34-2.59) (eFigure 9 in the Supplement). Of the studies included only in the qualitative synthesis, Maksimycheva et al^45^ and Da Silva Garrote et al^32^ found that underweight is associated with higher odds for *P aeruginosa* colonization, whereas 3 studies did not find significant differences between BMI and FFM categories.^23,31,51^

The studies by Stephenson et al,^52^ Bonhoure et al,^27^ and Harindhanavudhi et al^38^ were identified as showing significance regarding PI and CFRD by the leave-1-out sensitivity analysis (eFigures 10-13 in the Supplement). Funnel plots were created, and the Egger test was performed to detect publication bias. There was no small-study effect found in our analyses (eFigure 14 and eFigure 15 in the Supplement).

### Quality Assessment of Studies

Regarding FEV_1_%, 23% of the eligible studies (3 of 13) were assessed to be high risk and 46% (6 of 13) as moderate risk. High risk was shown for PI (56% [5 of 9]) and CFRD (57% [4 of 7]). Detailed results of risk of bias assessments are shown in eFigures 16 to 24 in the Supplement.

## Discussion

Our results suggest that higher BMI is associated with favorable clinical outcomes in patients with CF. Both overweight and obesity are associated with clinically significantly better pulmonary function compared with normal weight. A possible explanation could be the higher proportion of FFM in individuals who are overweight, which is associated with higher FEV_1_% and physical well-being.^35,40,49,55,56^ It has also been reported that patients with CF who are overweight have markedly fewer exacerbations that could contribute to loss of appetite.^38^ Moudiou et al^57^ described a negative correlation between resting energy expenditure (REE) and BMI *z* score. Resting energy expenditure is the amount of energy that is necessary to maintain basic body functions, such as digestion and breathing, and requires approximately 60% of the total calorie need.^58^ The worse the condition of the lungs, the higher the level of REE. Moreover, patients with PI were reported to have a higher REE compared with those with sufficient pancreatic function.^5,6,58^ Based on these data, we hypothesized that excess weight could cover the increased energy requirement during chronic inflammation.^56^ The favorable effect of weight gain has also been visible in the past few years as modulator therapy has become available as a novel treatment in CF.^59^ The continuous spread of modulators that aim to diminish the influence of *CFTR* dysfunction has been shown to have beneficial effect on body composition.^60^ In addition to weight gain, the proportion of FFM is increasing, which is associated with the reduction of REE.

Although we intended to include studies that compared patients with CF based not only on BMI but including FFM and FM, there were not enough eligible studies examining FFM and FM for a quantitative synthesis to be performed. All studies included in the qualitative synthesis highlighted the importance of the FFM proportion that leads to better pulmonary function. However, the influence of higher FM on FEV_1_% is not obvious. Alvarez et al^61^ reported that FEV_1_% is inversely associated with FM, whereas Panagopoulou et al^48^ described a correlation between body fat percentage and pulmonary function.

Our results regarding the association between higher BMI and the lower odds for PI are consistent with previous research.^27,48,52^ In patients with sufficient exocrine pancreatic function, adequate digestion and absorption are more likely to be present and can lead to higher BMI.

We found underweight to be associated with a higher prevalence of CFRD, which is in accordance with general clinical observations. In patients with CF who are underweight, there are several associated risk factors that contribute to the development of CFRD, such as PI or insulinopenia.^62^ We assumed that insulin resistance, as the minor component of CFRD, becomes more dominant in individuals who are overweight or obese and may lead to higher odds for diabetes compared with those of normal BMI. However, our results did not confirm this hypothesis, showing significantly lower odds for CFRD in patients with a BMI greater than or equal to 25.

Although our results showed that BMI greater than or equal to 25 does not have a statistically relevant association with glucose homeostasis (fasting glucose, fasting insulin, and HbA_1c_ levels), overweight and obesity are associated with higher total cholesterol and triglyceride levels. However, none of these elevated values exceeded the upper limit of normal.

Based on our results, the higher the BMI, the better the investigated clinical indices; we found no obvious evidence to be associated with harmful effects. However, the assessment of FFM and FM could provide more precise information. Several studies emphasized the potential usefulness of FFM as a more detailed assessment of body composition compared with BMI. The prevalence of hidden FFM depletion (FFM<5th percentile and normal BMI) is unexpectedly high (10%-20%) among patients with CF and is associated with increased disease severity, including reduced lung function, frequent pulmonary exacerbations, and increased inflammation.^31,35,40,55^ Therefore, measuring body composition in patients with CF may be more informative than the single use of BMI as an indicator of optimal health and nutritional status.

### Strengths and Limitations

To our knowledge, this is the first systematic review and meta-analysis assessing body composition in patients with CF in detail, including 3100 patients, with special focus on those who are overweight and obese. Moreover, we performed a meta-analysis regarding 9 outcomes, and subgroup analyses were performed for 3 outcomes.

Our study has limitations. There was substantial heterogeneity in the comparison of patients with normal weight and those who are obese regarding pulmonary function, and the source of substantial heterogeneity could not be identified by subgroup analysis. Furthermore, most of the studies did not report transplant status; thus, we could not perform subgroup analysis, and none of the pediatric studies reported the measurement method of respiratory function in children younger than 6 years.

## Conclusions

Our findings suggest that nutritional status plays an important role in maintaining organ function in patients with CF. Because we noted that a higher BMI is associated with better clinical parameters, we advise clinicians to reconsider increasing the currently recommended target BMI (22 for women and 23 for men). The use of a nutritional strategy that increases BMI, at least until the upper limit of normal BMI is reached, should be included in the daily protocol. Our results suggest that careful evaluation of body composition (FFM and FM) should be incorporated into everyday clinical practice. Studies with long-term follow-up are required to investigate the possible harmful effects of higher BMI, higher FM, and high-fat diet. Further observational studies are necessary focusing on major components of body composition (FFM and FM) with BMI.

## Notes

Supplement. **eMethods.** Search Strategy and Selection **eFigure 1.** Forest Plot Showing Pulmonary Function in Adults, Children, and Mixed Patient Population in the Comparison of Underweight and Non-Underweight Patients **eFigure 2.** Forest Plot Displaying the Risk for Exocrine Insufficiency in Subgroup Analysis in the Comparison of Underweight and Non-Underweight Patients **eFigure 3.** Forest Plot Displaying the Risk for CF-Related Diabetes in the Comparison of Underweight and Non-Underweight Patients **eFigure 4.** Forest Plots Showing Fasting Glucose Level in the Comparison of Different BMI Categories **eFigure 5.** Forest Plots Showing HbA_1c_% Level in the Comparison of Different BMI Categories **eFigure 6.** Forest Plot Displaying Fasting Insulin Level in the Comparison of Underweight and Non-Underweight Patients **eFigure 7.** Forest Plots Showing Total Cholesterol Level in the Comparison of Different BMI Categories **eFigure 8.** Forest Plots Showing Triglyceride Level in the Comparison of Different BMI Categories **eFigure 9.** Forest Plots Showing *Pseudomonas aeruginosa* Colonization in the Comparison of Different BMI Categories **eTable.** Table of Correlation Coefficients **eFigure 10.** Leave-1-Out Sensitivity Analysis Regarding Pulmonary Function in the Comparison of Different BMI Categories **eFigure 11.** Leave-1-Out Sensitivity Analysis Regarding Exocrine Insufficiency in the Comparison of Normal Weight and Underweight Patients **eFigure 12.** Leave-1-Out Sensitivity Analysis Regarding CF-Related Diabetes in the Comparison of Normal Weight and Underweight Plus Obese Groups **eFigure 13.** Leave-1-Out Sensitivity Analysis Regarding CF-Related Diabetes in Subgroup Analysis in the Comparison of Underweight and Non-Underweight Patients **eFigure 14.** Funnel Plot of the Included 8 Studies Regarding Pulmonary Function in the Comparison of Normal Weight and Overweight Patients **eFigure 15.** Funnel Plot of the Included 12 Studies Regarding Pulmonary Function in Subgroup Analysis in the Comparison of Underweight and Non-Underweight Patients **eFigure 16.** Risk of Bias Assessment at Study and Domain Level Regarding Pulmonary Function (FEV_1_%) **eFigure 17.** Risk of Bias Assessment at Study and Domain Level Regarding Exocrine Insufficiency **eFigure 18.** Risk of Bias Assessment at Study and Domain Level Regarding CFRD **eFigure 19.** Risk of Bias Assessment at Study and Domain Level Regarding Fasting Glucose **eFigure 20.** Risk of Bias Assessment at Study and Domain Level Regarding HbA_1c_% **eFigure 21.** Risk of Bias Assessment at Study and Domain Level Regarding Total Cholesterol **eFigure 22.** Risk of Bias Assessment at Study and Domain Level Regarding Triglycerides **eFigure 23.** Risk of Bias Assessment at Study and Domain Level Regarding *Pseudomonas aeruginosa* Colonization **eFigure 24.** Risk of Bias Assessment at Study and Domain Level Regarding Fasting Insulin
eReferences
