# Supplementary material for: Association of Body Mass Index With Clinical Outcomes in Patients With Cystic Fibrosis: A Systematic Review and Meta-analysis
Source: JAMA Netw Open. 2022 Mar 7;5(3):e220740. doi: 10.1001/jamanetworkopen.2022.0740 (PMC8902650; doi:10.1001/jamanetworkopen.2022.0740)
Supplement: Supplement. — eMethods. Search Strategy and Selection eFigure 1. Forest Plot Showing Pulmonary Function in Adults, Children, and Mixed Patient Population in the Comparison of Underweight and Non-Underweight Patients eFigure 2. Forest Plot Displaying the Risk for Exocrine Insufficiency in Subgroup Analysis in the Comparison of Underweight and Non-Underweight Patients eFigure 3. Forest Plot Displaying the Risk for CF-Related Diabetes in the Comparison of Underweight and Non-Underweight Patients eFigure 4. Forest Plots Showing Fasting Glucose Level in the Comparison of Different BMI Categories eFigure 5. Forest Plots Showing HbA1c% Level in the Comparison of Different BMI Categories eFigure 6. Forest Plot Displaying Fasting Insulin Level in the Comparison of Underweight and Non-Underweight Patients eFigure 7. Forest Plots Showing Total Cholesterol Level in the Comparison of Different BMI Categories eFigure 8. Forest Plots Showing Triglyceride Level in the Comparison of Different BMI Categories eFigure 9. Forest Plots Showing Pseudomonas aeruginosa Colonization in the Comparison of Different BMI Categories eTable. Table of Correlation Coefficients eFigure 10. Leave-1-Out Sensitivity Analysis Regarding Pulmonary Function in the Comparison of Different BMI Categories eFigure 11. Leave-1-Out Sensitivity Analysis Regarding Exocrine Insufficiency in the Comparison of Normal Weight and Underweight Patients eFigure 12. Leave-1-Out Sensitivity Analysis Regarding CF-Related Diabetes in the Comparison of Normal Weight and Underweight Plus Obese Groups eFigure 13. Leave-1-Out Sensitivity Analysis Regarding CF-Related Diabetes in Subgroup Analysis in the Comparison of Underweight and Non-Underweight Patients eFigure 14. Funnel Plot of the Included 8 Studies Regarding Pulmonary Function in the Comparison of Normal Weight and Overweight Patients eFigure 15. Funnel Plot of the Included 12 Studies Regarding Pulmonary Function in Subgroup Analysis in the Comparison of Underweight and Non-Underweight [file jamanetwopen-e220740-s001.pdf]

Supplementary Online Content

Nagy R, Gede N, Ocskay K, et al. Association of body mass index with clinical outcomes in patients with cystic fibrosis: a systematic review and meta-analysis. *JAMA Netw Open*. 2022;5(3):e220740. doi:10.1001/jamanetworkopen.2022.0740

eMethods. Search Strategy and Selection

eFigure 1. Forest Plot Showing Pulmonary Function in Adults, Children, and Mixed Patient Population in the Comparison of Underweight and Non-Underweight Patients

eFigure 2. Forest Plot Displaying the Risk for Exocrine Insufficiency in Subgroup Analysis in the Comparison of Underweight and Non-Underweight Patients

eFigure 3. Forest Plot Displaying the Risk for CF-Related Diabetes in the Comparison of Underweight and Non-Underweight Patients

eFigure 4. Forest Plots Showing Fasting Glucose Level in the Comparison of Different BMI Categories

eFigure 5. Forest Plots Showing HbA_{1c}% Level in the Comparison of Different BMI Categories

eFigure 6. Forest Plot Displaying Fasting Insulin Level in the Comparison of Underweight and Non-Underweight Patients

eFigure 7. Forest Plots Showing Total Cholesterol Level in the Comparison of Different BMI Categories

eFigure 8. Forest Plots Showing Triglyceride Level in the Comparison of Different BMI Categories

eFigure 9. Forest Plots Showing *Pseudomonas aeruginosa* Colonization in the Comparison of Different BMI Categories

eTable. Table of Correlation Coefficients

eFigure 10. Leave-1-Out Sensitivity Analysis Regarding Pulmonary Function in the Comparison of Different BMI Categories

eFigure 11. Leave-1-Out Sensitivity Analysis Regarding Exocrine Insufficiency in the Comparison of Normal Weight and Underweight Patients

eFigure 12. Leave-1-Out Sensitivity Analysis Regarding CF-Related Diabetes in the Comparison of Normal Weight and Underweight Plus Obese Groups

eFigure 13. Leave-1-Out Sensitivity Analysis Regarding CF-Related Diabetes in Subgroup Analysis in the Comparison of Underweight and Non-Underweight Patients

eFigure 14. Funnel Plot of the Included 8 Studies Regarding Pulmonary Function in the Comparison of Normal Weight and Overweight Patients

eFigure 15. Funnel Plot of the Included 12 Studies Regarding Pulmonary Function in Subgroup Analysis in the Comparison of Underweight and Non-Underweight Patients

eFigure 16. Risk of Bias Assessment at Study and Domain Level Regarding Pulmonary Function (FEV_1%)

eFigure 17. Risk of Bias Assessment at Study and Domain Level Regarding Exocrine Insufficiency

eFigure 18. Risk of Bias Assessment at Study and Domain Level Regarding CFRD

eFigure 19. Risk of Bias Assessment at Study and Domain Level Regarding Fasting Glucose

eFigure 20. Risk of Bias Assessment at Study and Domain Level Regarding HbA_{1c}%

eFigure 21. Risk of Bias Assessment at Study and Domain Level Regarding Total Cholesterol

eFigure 22. Risk of Bias Assessment at Study and Domain Level Regarding Triglycerides

eFigure 23. Risk of Bias Assessment at Study and Domain Level Regarding *Pseudomonas aeruginosa* Colonization

eFigure 24. Risk of Bias Assessment at Study and Domain Level Regarding Fasting Insulin

eReferences

This supplementary material has been provided by the authors to give readers additional information about their work.

© 2022 Nagy R et al. *JAMA Network Open.*

eMethods

Search strategy and selection

The literature search was conducted on November 2, 2020, in three databases: MEDLINE (via PubMed), Embase, and Cochrane Central Register of Controlled Trials (CENTRAL). The following search key was used: ("cystic fibrosis" OR mucoviscidosis) AND ("body fat" OR bmi OR "body mass" OR "body composition" OR "fat free mass" OR "muscle mass" OR "lean body mass" OR adiposity OR obes* OR "body weight" OR overweight OR sarcopen*), without any restrictions.

The following data were extracted from each eligible article: study name, first author, publication year, Digital Object Identifier (DOI), recruitment period, gender distribution, age distribution, genotype, patient number and mean or median values of outcomes of interest. Correlation coefficients were also extracted regarding the association of BMI or body composition and clinical outcomes.

© 2022 Nagy R et al. *JAMA Network Open.*

eFigure 1. **Forest plot showing pulmonary function in adults, children and mixed patient population in the comparison of underweight and non-underweight patients.**

Note: substantial heterogeneity can be detected.

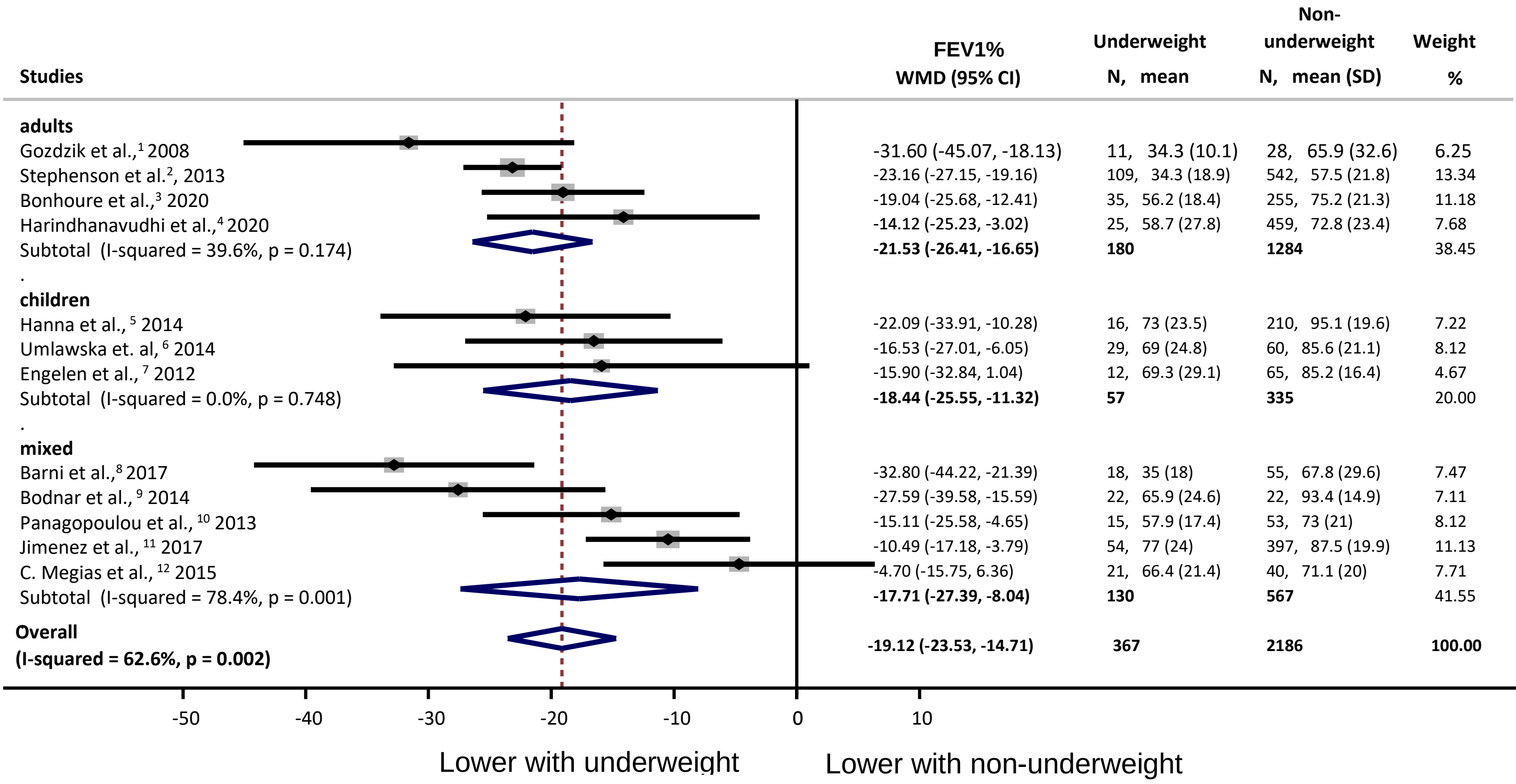

© 2022 Nagy R et al. *JAMA Network Open.*

eFigure 2. **Forest plot displaying the risk for exocrine insufficiency in subgroup analysis in the comparison of underweight and non-underweight patients**.

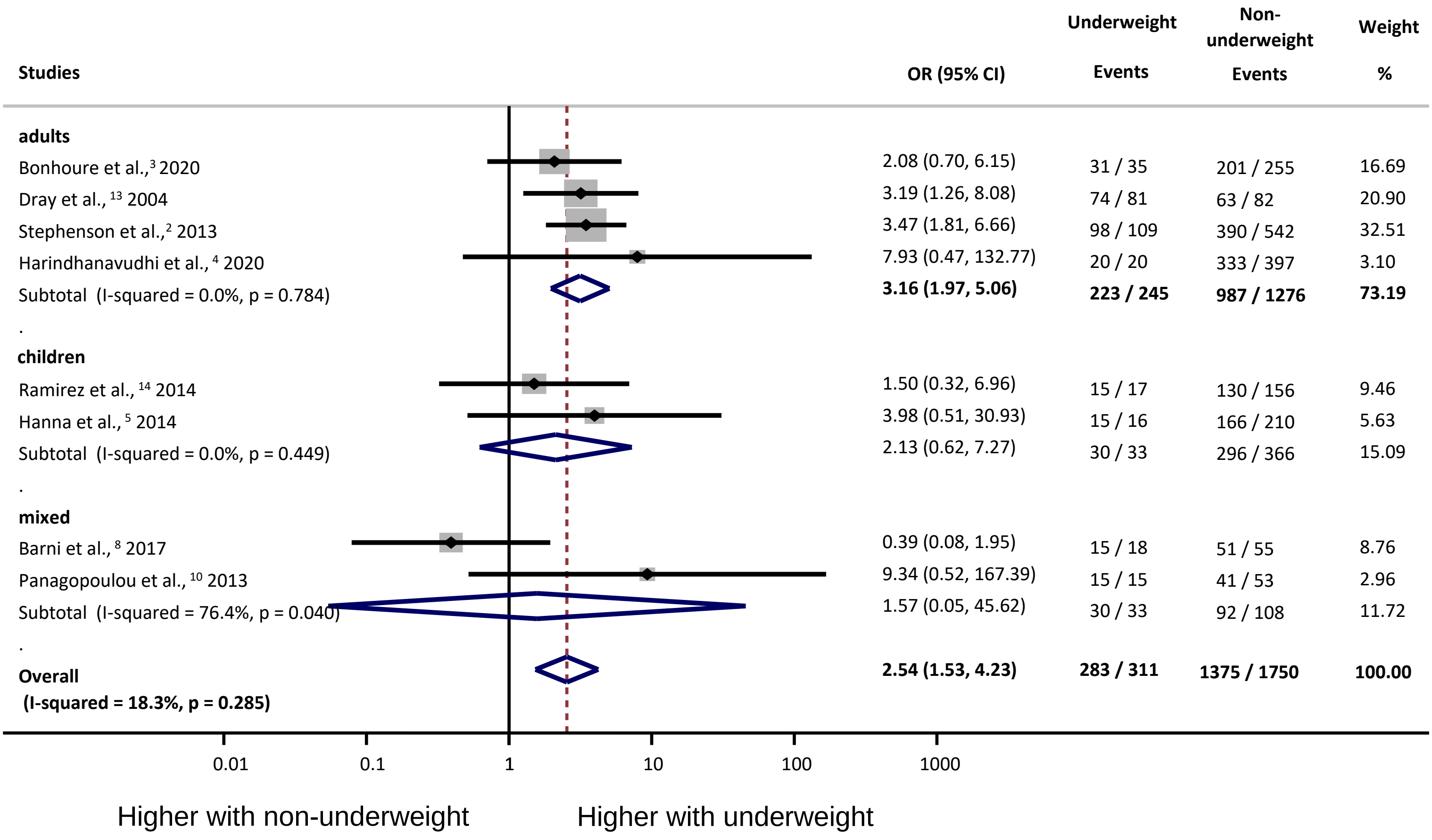

© 2022 Nagy R et al. *JAMA Network Open.*

eFigure 3. - Forest plot displaying the risk for CF-related diabetes in the comparison of underweight and non-underweight.

Studies	OR (95% CI)	Underweight Events	Non-underweight Events	Weight %
adults				
Stephenson et al.[2], 2013	1.13 (0.52, 2.45)	27 / 109	12 / 53	23.34
Dray et al.,[13] 2004	1.41 (0.63, 3.13)	17 / 81	13 / 82	22.09
Harindhanavudhi et al.,[4] 2020	1.82 (0.80, 4.14)	15 / 25	206 / 456	20.87
Bonhoure et al.[3,] 2020	1.86 (0.78, 4.43)	8 / 35	35 / 255	18.77
Subtotal (I-squared = 0.0%, p = 0.800)	**1.50 (1.00, 2.25)**	**67 / 250**	**266 / 846**	**85.08**
.				
mixed				
Barni et al.,[8] 2017	0.73 (0.14, 3.82)	2 / 18	8 / 55	5.17
Panagopoulou et al.,[10] 2013	2.99 (0.90, 9.94)	7 / 15	12 / 53	9.75
Subtotal (I-squared = 45.3%, p = 0.176)	1.67 (0.43, 6.50)	9 / 33	20 / 108	14.92
.				
**Overall (I-squared = 0.0%, p = 0.705)**	**1.55 (1.06, 2.25)**	**76 / 283**	**286 / 954**	**100.00**

NOTE: Weights are from random effects analysis

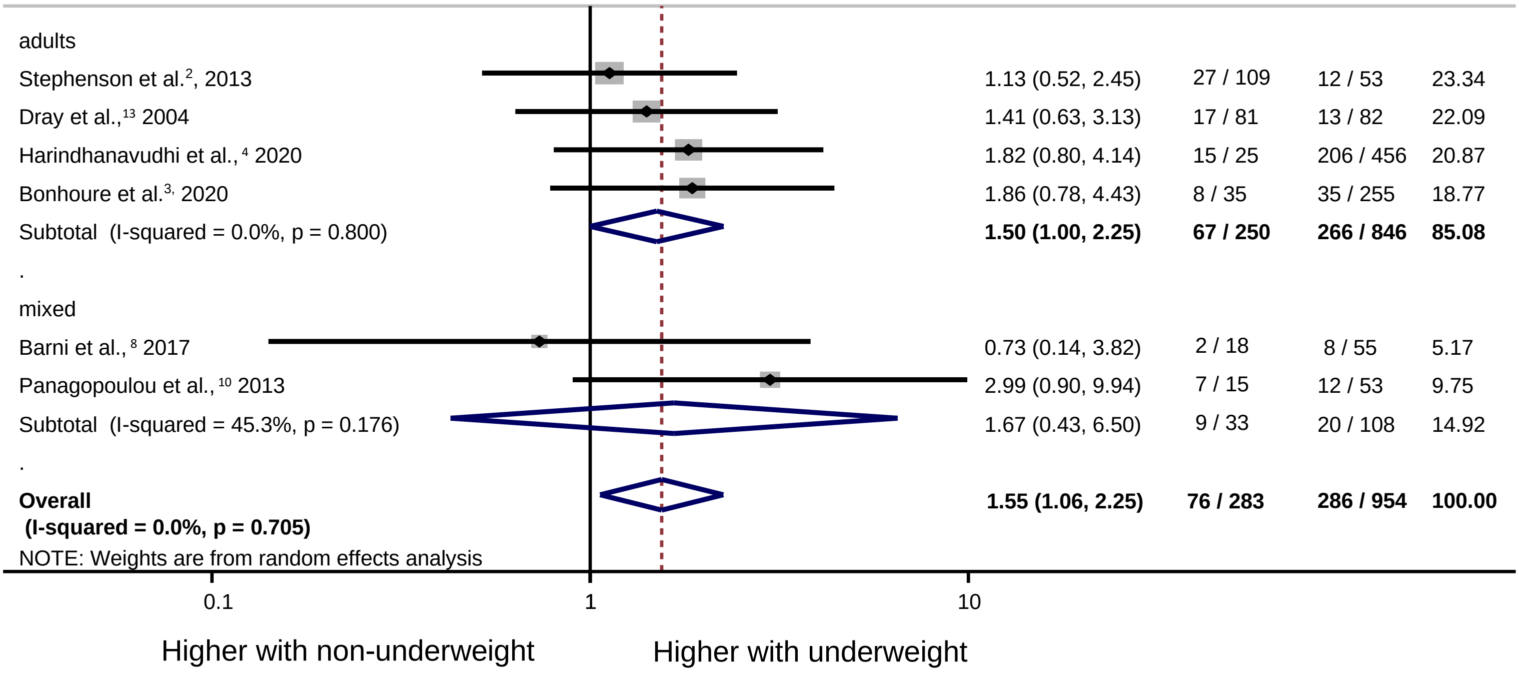

© 2022 Nagy R et al. *JAMA Network Open.*

eFigure 4. - Forest plots showing fasting glucose level in the comparison of different BMI categories.

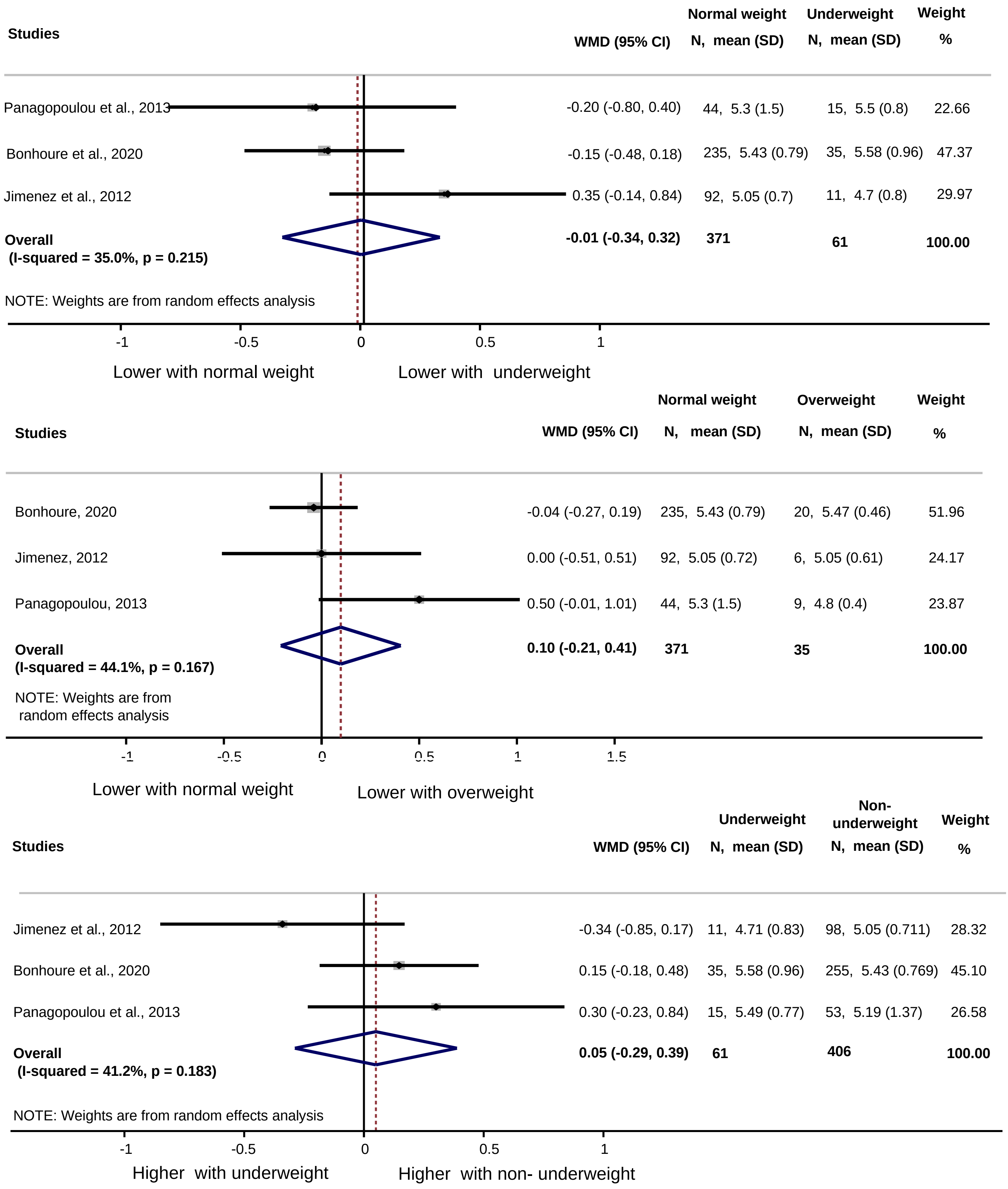

© 2022 Nagy R et al. *JAMA Network Open*.

eFigure 5. **Forest plots showing HbA1c% level in the comparison of different BMI categories**

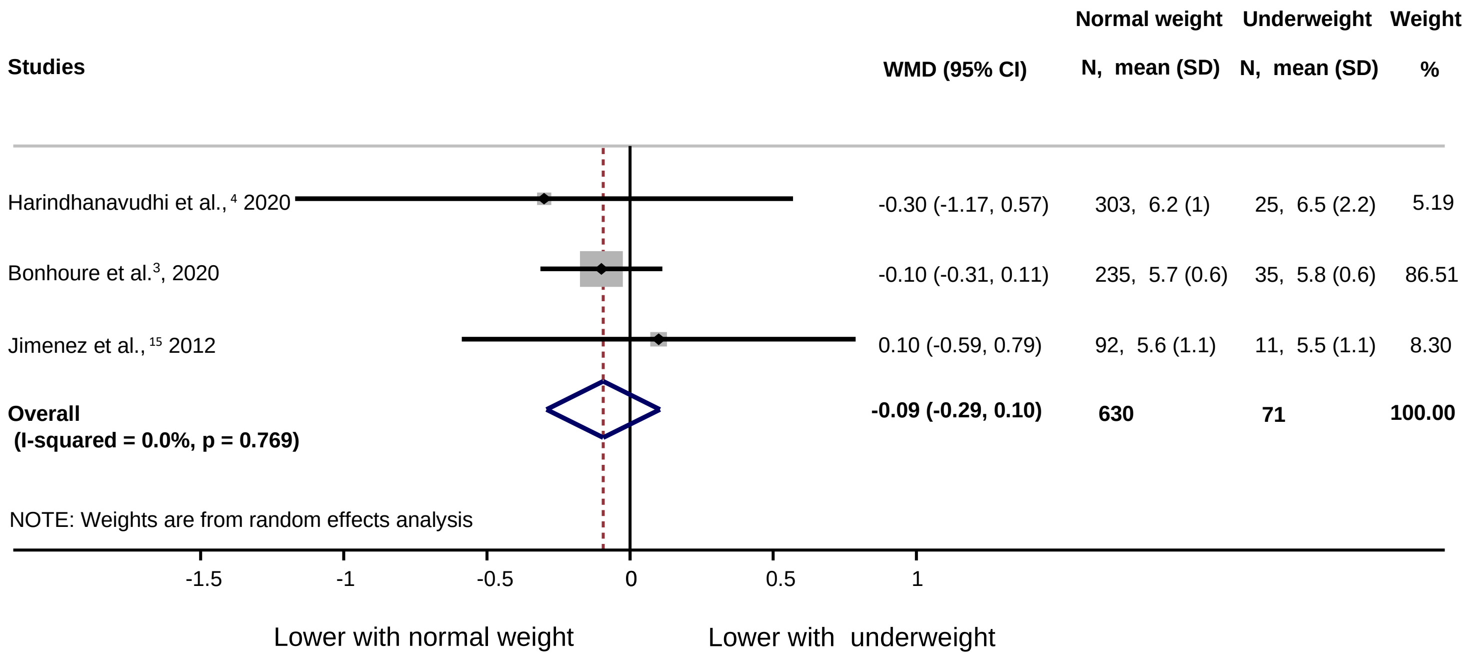

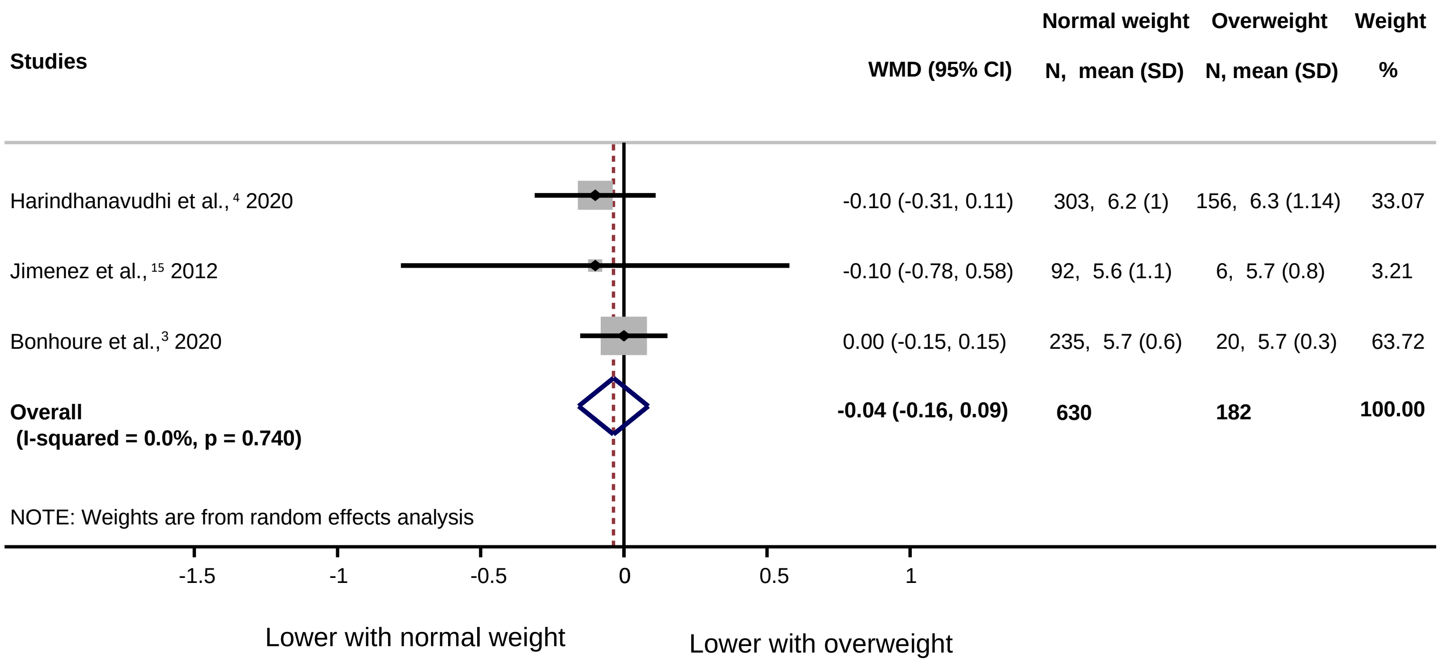

© 2022 Nagy R et al. *JAMA Network Open.*

eFigure 6. **Forest plot displaying fasting insulin level in the comparison of underweight and non-underweight patients**.

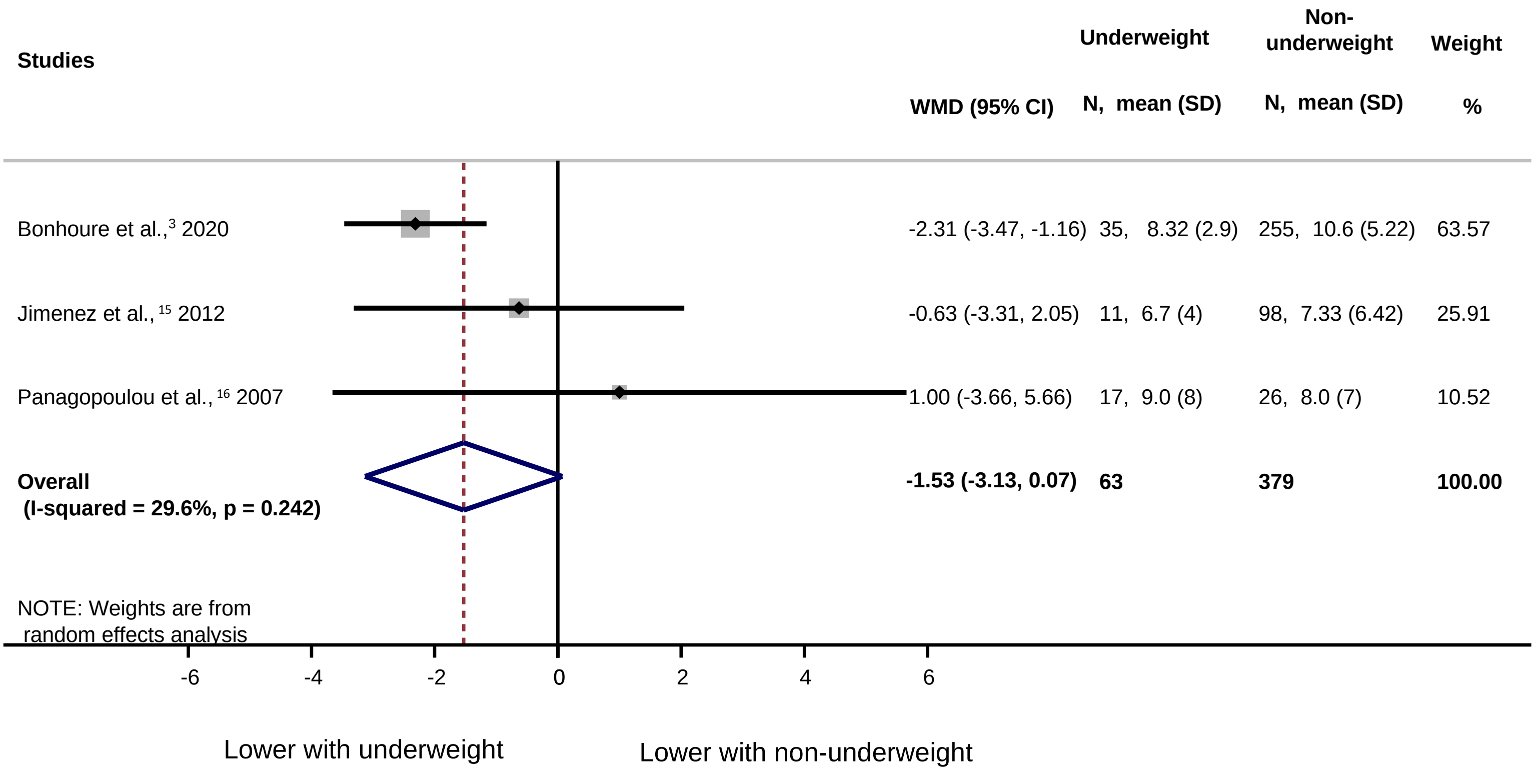

© 2022 Nagy R et al. *JAMA Network Open.*

eFigure 7. **Forest plots showing total cholesterol level in the comparison of different BMI categories.**

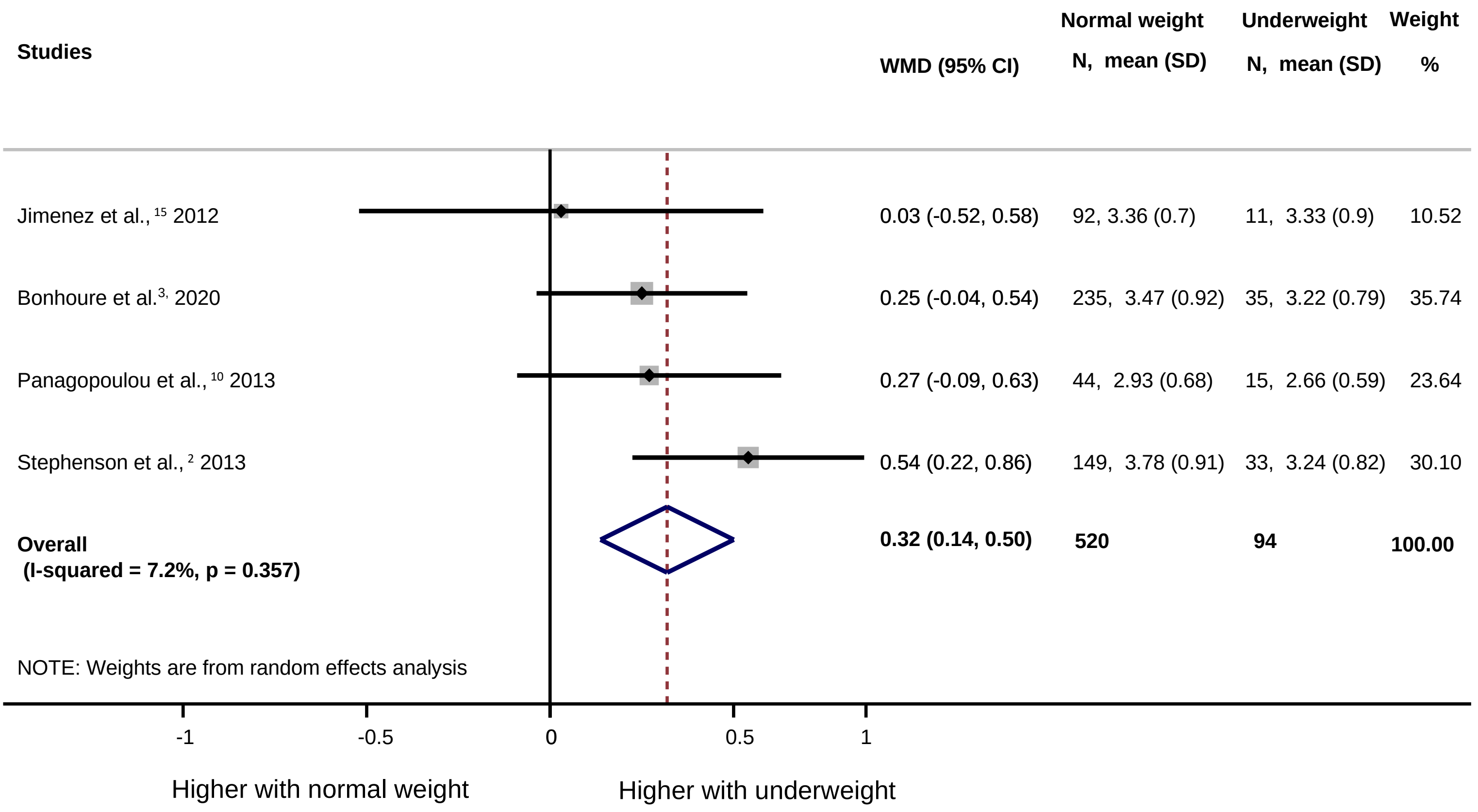

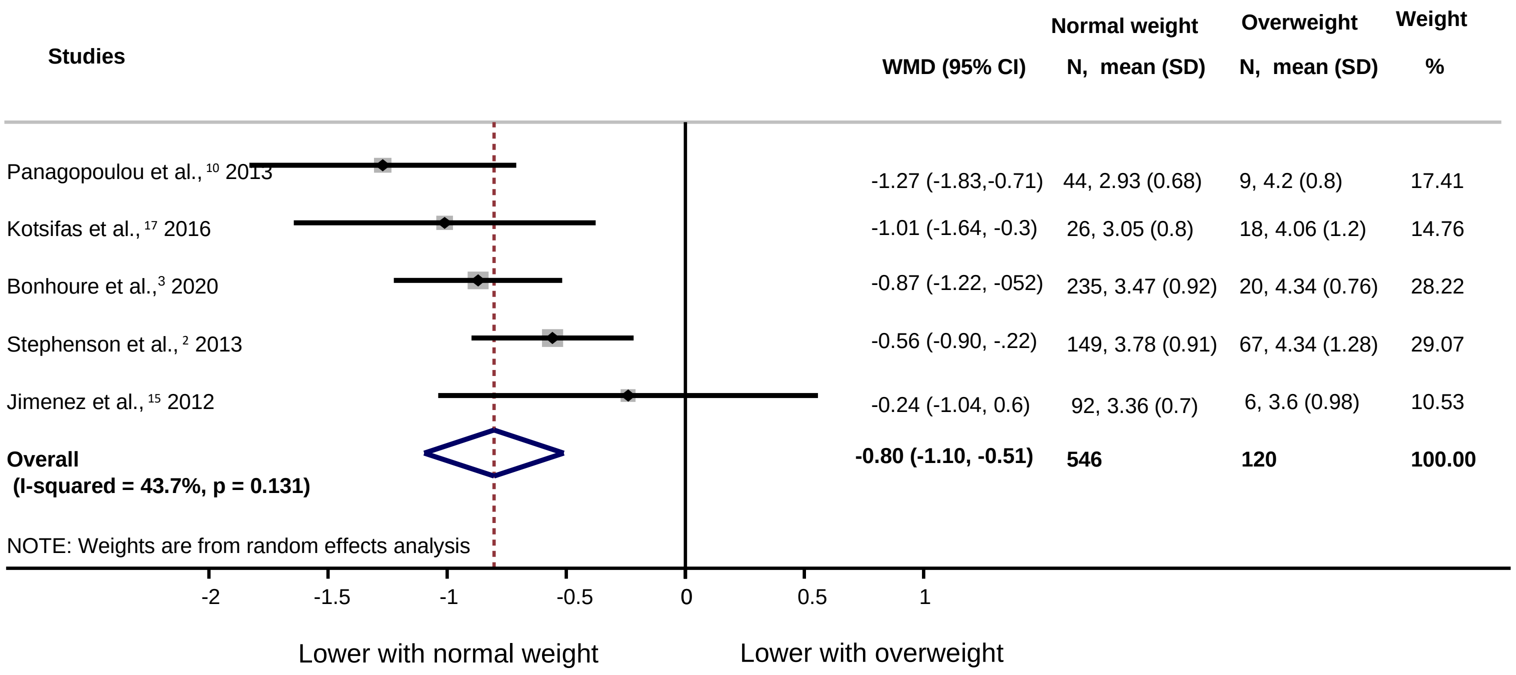

© 2022 Nagy R et al. *JAMA Network Open.*

eFigure 8. **Forest plots showing triglyceride level in the comparison of different BMI categories.**

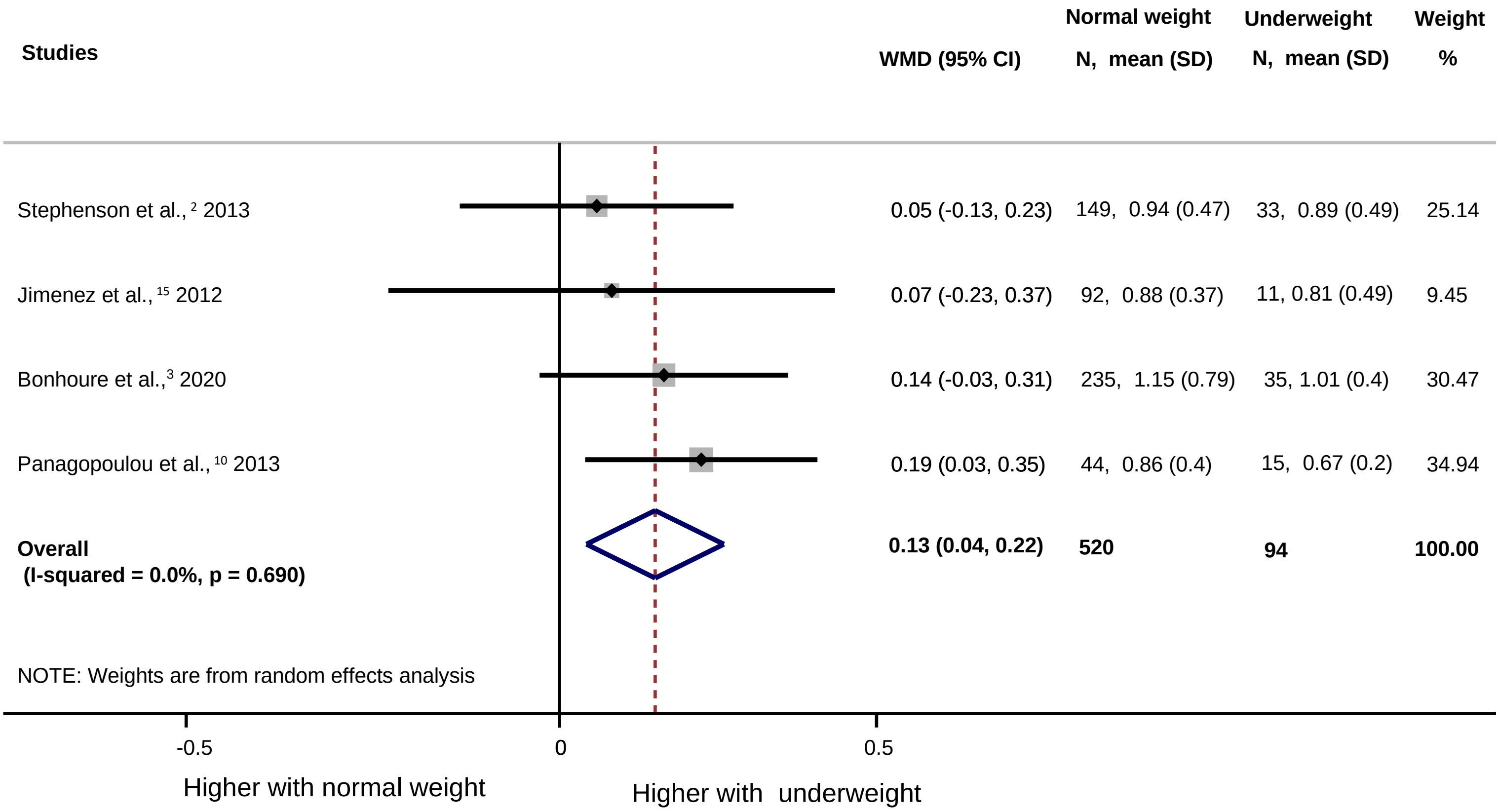

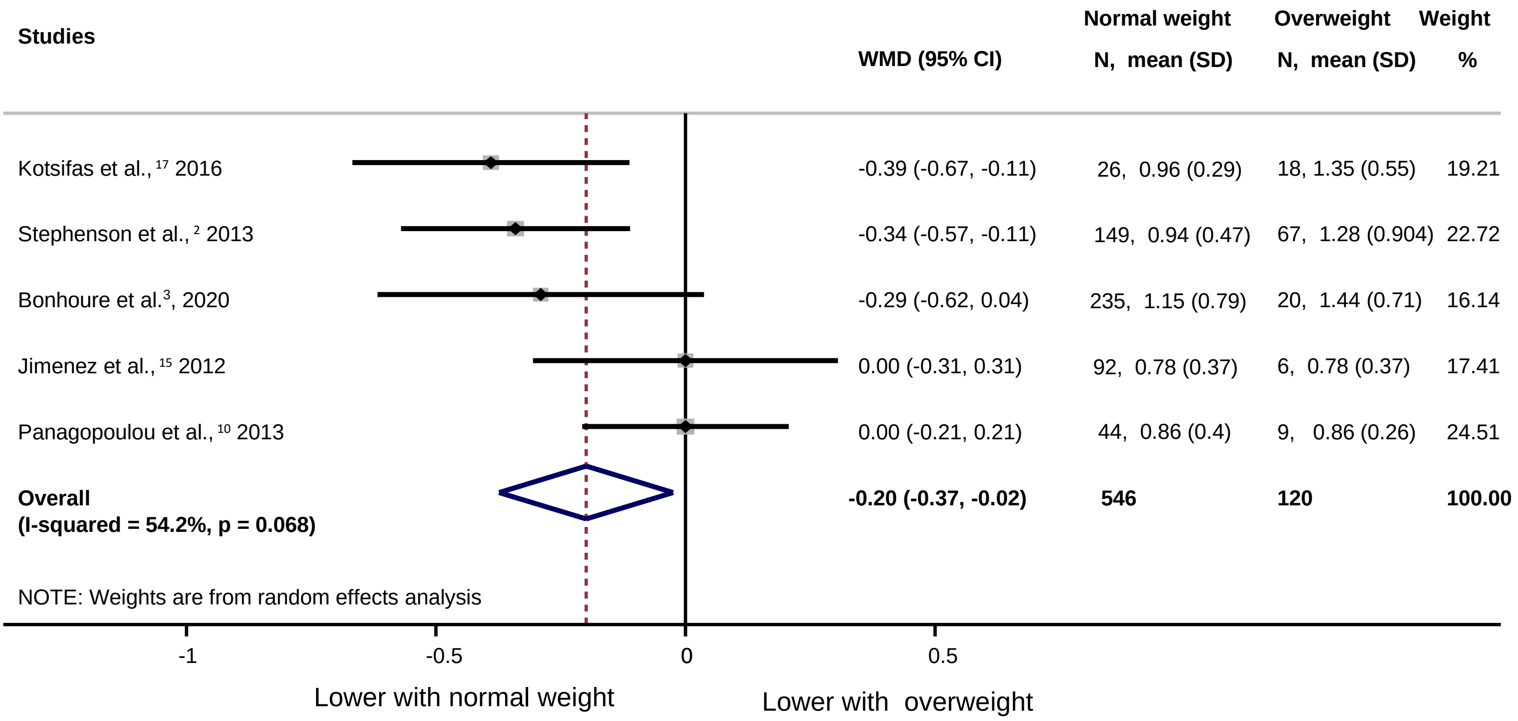

© 2022 Nagy R et al. *JAMA Network Open.*

eFigure 9. **Forest plot showing *Pseudomonas aeruginosa* colonization in the comparison of underweight and non-underweight**.

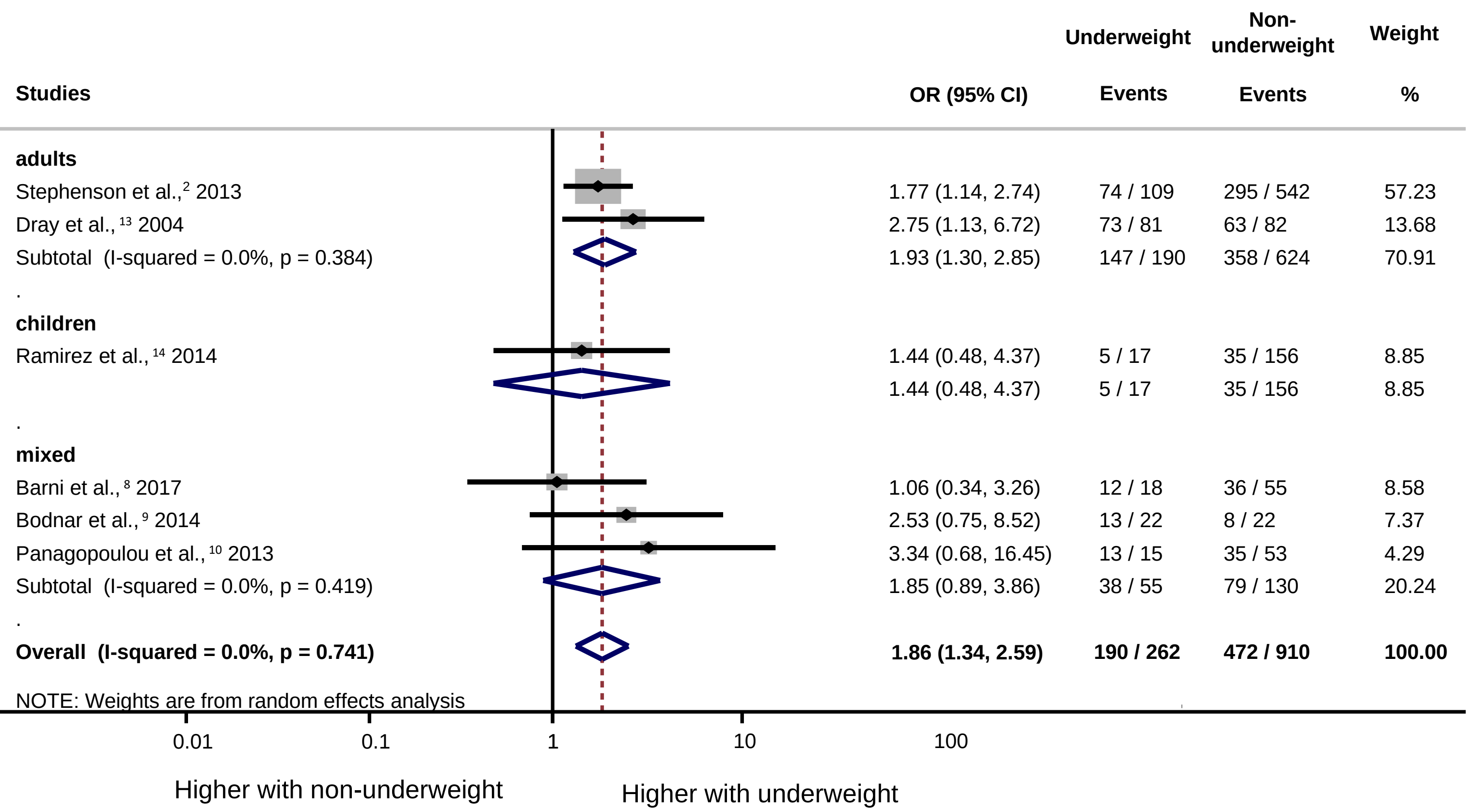

© 2022 Nagy R et al. *JAMA Network Open.*

eTable. Table of correlation coefficients.

Author	Parameters	r-value	p-value	Patient number	Age group
Barja et al.[18]	FEV1% - BMI z score	0.459	0.02	25	children
Pedreira et al. [19]	FEV1% - BMI z-score	0.59	0.0001	50	children
Asseiceire et al.[20*]	FEV1% - BMI z-score	0.414	0.026	34	children
Junge et al.[21*]	FEV1% - BMI z-score	0.326	<0.001	156	children
Mexia et al.[22*]	FEV1% - BMI z-score	0.530	0.002	39	children
Cherniak et al. [23]	FEV1% - BMI z-score	0.47	< 0.001	291	mixed
Vukic et al.[24*]	FEV1% - BMI	0.31	0.09	30	adults
Bell et al. [25]	FEV1% - BMI	0.51	<0.0001	81	adult
Gozdzik et al.[1]	FEV1% - BMI	0.34	≤0.03	39	adult
Callela et al. [26]	FEV1% - BMI	0.52	<0.001	96	children
Coderre et al. [27]	FEV1% - BMI	0.32	<0.01	187	adult
Dray et al.	et al.FEV1% - BMI	0.46	<0.001	163	adult
Er et al. [28]	FEV1% - BMI	0.46	< 0.001	76	adults
Ionescu et al. [29]	FEV1% - BMI	0.30	<0.02	56	adult
Jarad et al. [30]	FEV1% - BMI	0.42	< 0.0001	34	adult
Belson et al.[31*]	FEV1% - BMI	0.23	<0.005	190	adults
Cardenas et al. [32*]	FEV1% - BMI	0.35	0.001	96	adults
Bianchi et al. [33]	FEV1% - BMI	0.30	non-sign.	136	mixed
Moco et al. [34]	FEV1% - BMI	0.1	non-sign.	21	adults
Spirevska et al. [35*]	FEV1% - BMI	0.37	NA	49	mixed
Papalexopoulou et al. [36]	FEV 1% z-score - BMI z-score	0.097	0.616	29	mixed
Panagopoulou et al.[10]	FEV1% - BMI-SDS	0.498	0.000	68	mixed
Souza et al. [38]	FEV1% - BMIp	0.312	0.004	85	children
Engelen et al.[7]	FEV1% - FFMI %	0.39	< 0.001	77	mixed
Bolton et al. [37]	FEV1% - FFMI	0.418	0.003	511	adults
Ionescu et al. [29]	FEV1% - FFM	0.44	< 0.01	56	adults
Bianchi et al. [33]	FEV1% - FFM	0.61	< 0.01	136	mixed
Moco et al. [34]	FEV1% - FFM	0.2	non-sign.	21	adults
Asseiceire et al. [20*]	FEV1% - FFMI	0.413	0.045	34	children
Bisogno et al.[1*]	FEV1% - FFMI	0.55	<0.0001	85	adults
Vukic et al. [24*]	FEV1% - FFMI	0.52	0.0002	30	adults
King et al.[39]	FEV1% - FFMI z-score	0.41	<0.0001	86	adults
Sermet et al. [40]	FEV1% - FFM z -score	0.41	0.001	114	children
Pedreira et al. [19]	FEV1% - FFM z -score	0.30	0.03	50	children

© 2022 Nagy R et al. *JAMA Network Open.*

Callela et al. [26]	FEV1% - LBM	0.68	<0.001	96	children
Rochat et al.[41]	FEV 1%- T-LBM	0.847	0.0005	12	adults
Sripalan et al.[42]*	FEV 1%- T-LBM	0.39	0.04	34	adults
Papalexopoulou et al. [36]	FEV1% z-score - FFMI	0.299	0.115	29	mixed
Boat et al.[43]*	FEV1% - LMI	0.676	0.011	13	mixed
Panagopoulou et al.[10]	FEV1% - BF%	0.349	0.023	68	mixed
Belson et al. [31]*	FEV1% - BF %	0.19	<0.05	190	adults
Sills et al.[44]*	absolute FEV1 - BF%	- 0.2	0.03	103	adults
Engelen et al.[7]	FEV1% - FMI %	0.30	< 0.01	77	mixed
Belson et al. [31]*	FEV1% - FM	0.18	<0.05	190	adults
Bellissimo et al.[45]*	FG - VFM	0.45	0.04	22	adults

r value indicates the strength of the kinear relationship between two variables. p value represents the probability that the correlation between the two variables occured by chance. * conference abstract

© 2022 Nagy R et al. *JAMA Network Open.*

eFigure 10. Leave-1-out sensitivity analysis regarding pulmonary function in the comparison of different BMI categories.

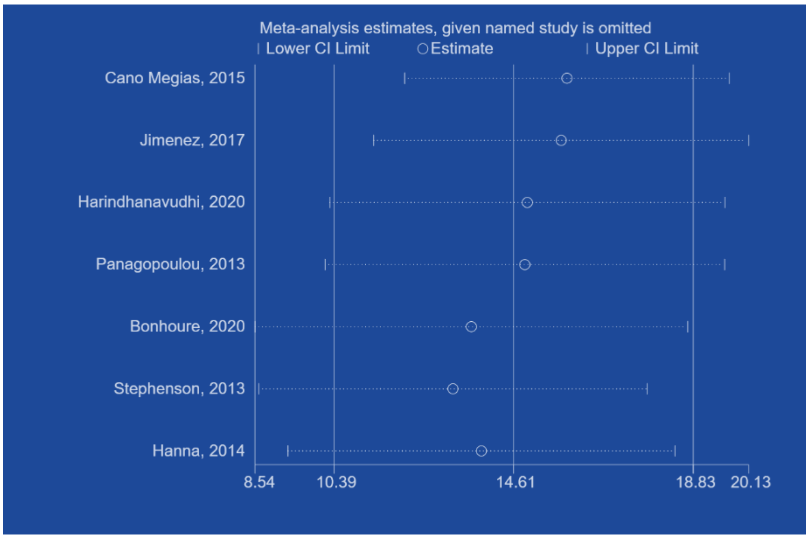

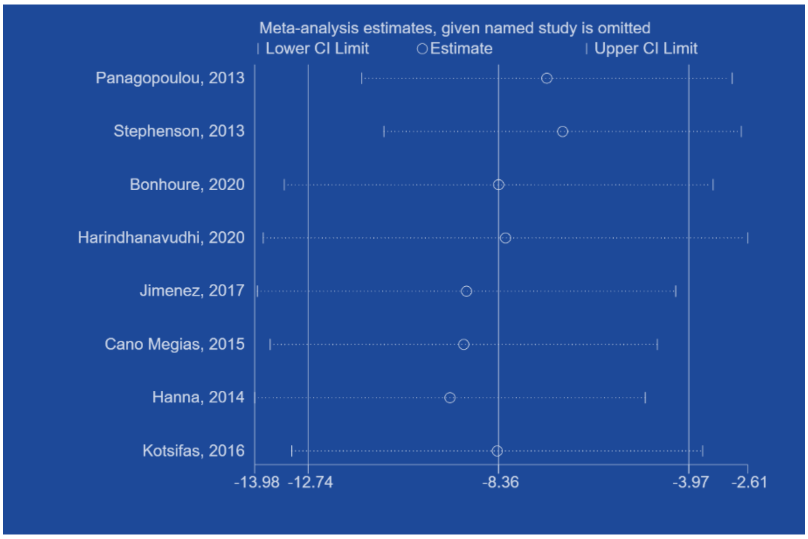

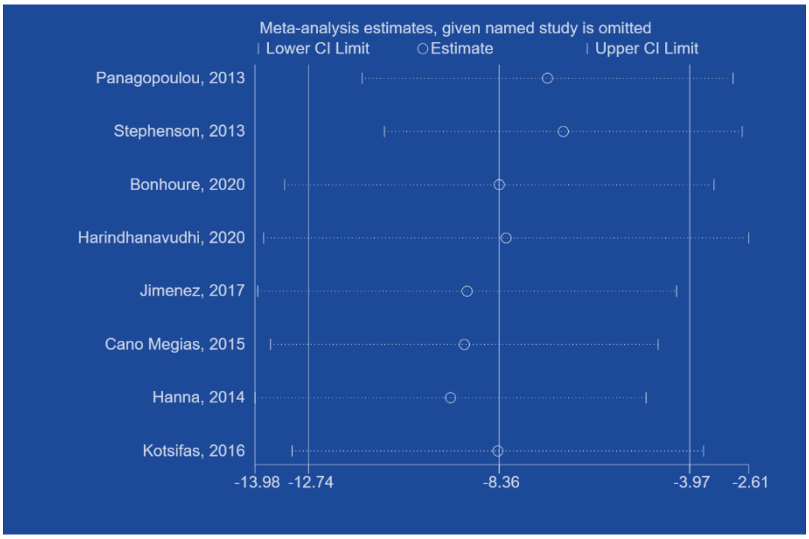

Omitting a study does not influence the result in any case.

© 2022 Nagy R et al. *JAMA Network Open.*

eFigure 11. Leave-1-out sensitivity analysis regarding exocrine insufficiency in the comparison of normal weight and underweight patients.

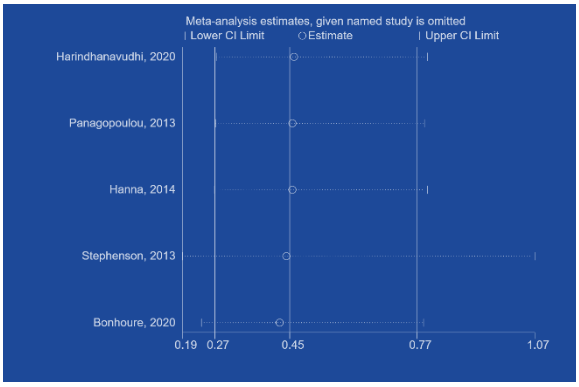

Leaving out the study of Stephenson will alter the result.

eFigure 12. Leave-1-out sensitivity analysis regarding CF-related diabetes in the comparison of normal weight and overweight plus obese groups.

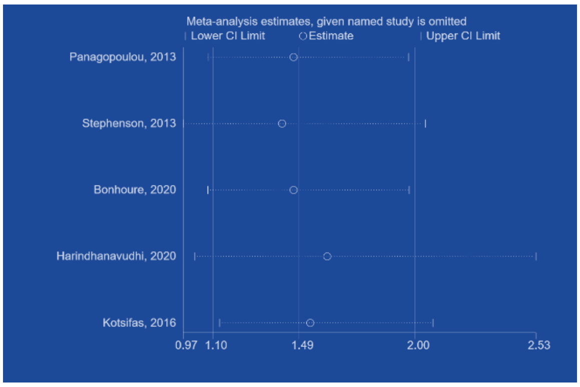

Leaving out the study of Stephenson et al.[2] will alter the significancy of the result.

© 2022 Nagy R et al. *JAMA Network Open.*

eFigure 13. Leave-1-out sensitivity analysis regarding CF-related diabetes in subgroup analysis in the comparison of underweight and non-underweight patients.

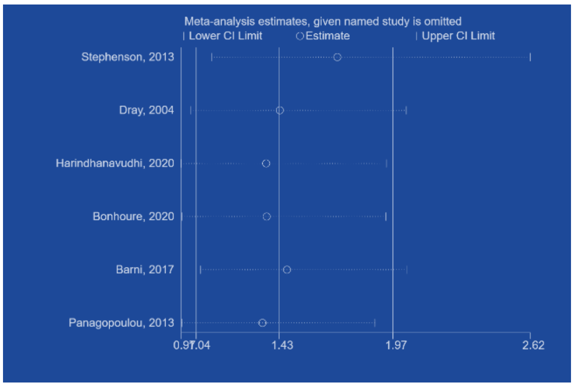

Leaving out the study of Bonhoure[3] and Harindhanavudhi[4] will alter the significancy of the result.

© 2022 Nagy R et al. *JAMA Network Open.*

eFigure 14. **Funnel plot of the included 8 studies regarding pulmonary function in the comparison of normal weight and overweight patients.**

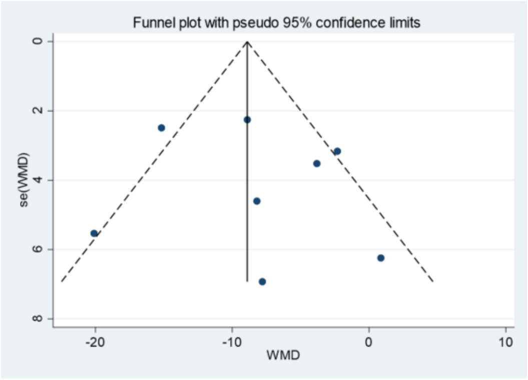

Egger's test: P = 0.650
(no small study effect)

The visual assessment and Egger's test (p>0.05) does not indicate asymmetry, thus publication bias is unlikely.

© 2022 Nagy R et al. *JAMA Network Open.*

eFigure 15. Funnel plot of the included 12 studies regarding pulmonary function in subgroup analysis in the comparison of underweight and non-underweight patients.

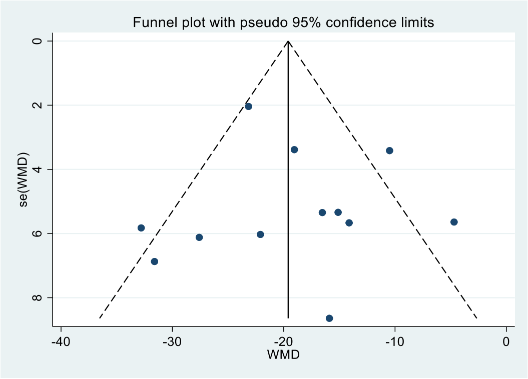

Egger's test: P = 0.751
(no small study effect)

The visual assessment and Egger's test (p>0.05) does not indicate asymmetry, thus publication bias is unlikely.

eFigure 16. **Risk of bias assessment at study and domain level regarding pulmonary function (FEV1%).**

Study	Study participation	Study attrition	Prognostic factor measurement	Outcome measurement	Study confounding	Statistical analysis reporting	**Overall risk of bias**	**Included in the MA**
**Panagopoulou et al., 2013[10]**	3	NA	3	2	2	3	2	yes
**Stephenson et al, 2013[2]**	3	NA	3	3	1	3	1	yes
**Bonhoure et al, 2020[3]**	3	NA	3	3	2	2	3	yes
**Harindhanavudhi et al.,2020[4]**	2	NA	3	3	1	3	1	yes
**Jimenez et al., 2017[11]**	3	NA	3	2	2	3	2	yes
**C. Megias et al.,2015[12]**	3	NA	3	3	2	2	2	yes
**Hanna et al., 2014[5]**	3	NA	3	2	2	3	2	yes
**Gozdzik et al.,2008[1]**	2	NA	3	3	2	3	2	yes
**Engelen et al.,2012[7]**	3	NA	3	3	2	3	3	yes
**Umlawska et al.,2014[6]**	2	NA	3	3	2	3	2	yes
**Barni et al.,2017[8]**	3	NA	3	3	3	3	3	yes
**Bodnar et al, 2014[9]**	3	NA	3	3	3	3	3	yes
**Kotsifas et al.,2016[17]**	2	NA	3	2	2	3	1	yes

Total	low	9		13	9	2	11	4
Total	moderate	4			4	9	2	6
Total	high					2		3
Total	NA		13					

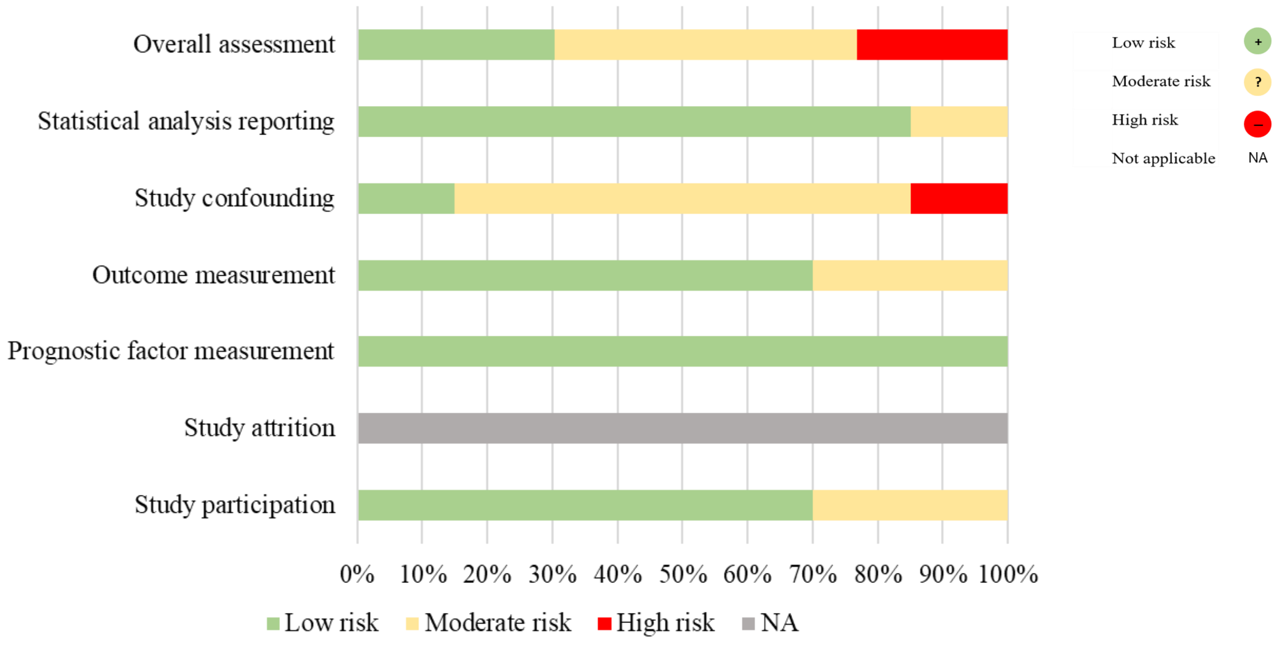

© 2022 Nagy R et al. *JAMA Network Open.*

eFigure 17. **Risk of bias assessment at study and domain level regarding exocrine insufficiency.**

Study	Study participation	Study attrition	Prognostic factor measurement	Outcome measurement	Study confounding	Statistical analysis reporting	**Overall risk of bias**	**Included in the MA**
**Panagopoulou et al., 2013[10]**	3	NA	3	3	2	3	3	yes
**Stephenson et al, 2013[2]**	3	NA	3	2	1	3	1	yes
**Bonhoure et al, 2020[3]**	3	NA	3	2	2	3	2	yes
**Harindhanavudhi et al.,2020[4]**	2	NA	3	2	1	3	1	yes
**Hanna et al., 2014[5]**	3	NA	3	2	2	3	2	yes
**Ramirez et al.,2014[14]**	2	NA	3	2	2	3	1	yes
**Barni et al.,2017[8]**	3	NA	3	3	3	3	3	yes
**Dray et al.,2004[13]**	2	NA	3	2	2	3	1	yes
**Kotsifas et al.,2016[17]**	2	NA	3	2	2	3	1	yes
Total low	5		9	3	1	9	2	
Total moderate	4			6	6		2	
Total high					2		5	
Total NA		9						

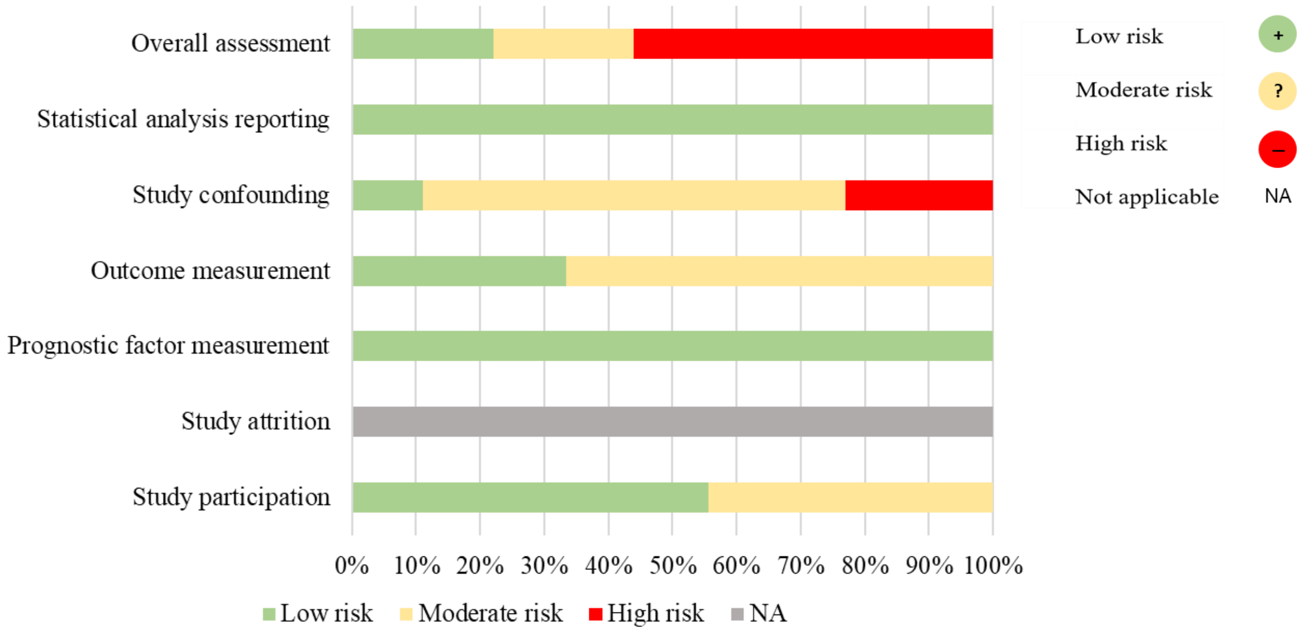

© 2022 Nagy R et al. *JAMA Network Open.*

eFigure 18. **Risk of bias assessment at study and domain levevel ragarding CFRD.**

Study	Study participation	Study attrition	Prognostic factor measurement	Outcome measurement	Study confounding	Statistical analysis reporting	**Overall risk of bias**	**Included in the MA**
**Panagopoulou et al., 2013[10]**	3	NA	3	3	2	3	3	yes
**Stephenson et al, 2013[2]**	3	NA	3	2	1	3	1	yes
**Bonhoure et al, 2020[3]**	3	NA	3	3	2	3	3	yes
**Harindhanavudhi et al.,2020[4]**	2	NA	3	3	1	3	1	yes
**Dray et al.,2004[13]**	2	NA	3	2	2	3	1	yes
**Barni et al.,2017[8]**	3	NA	3	2	3	3	2	yes
**Kotsifas et al.,2016[17]**	2	NA	3	2	2	3	1	yes
Total low	4		7	3	1	7	2	
Total moderate	3			4	4		1	
Total high					2		4	
Total NA		7						

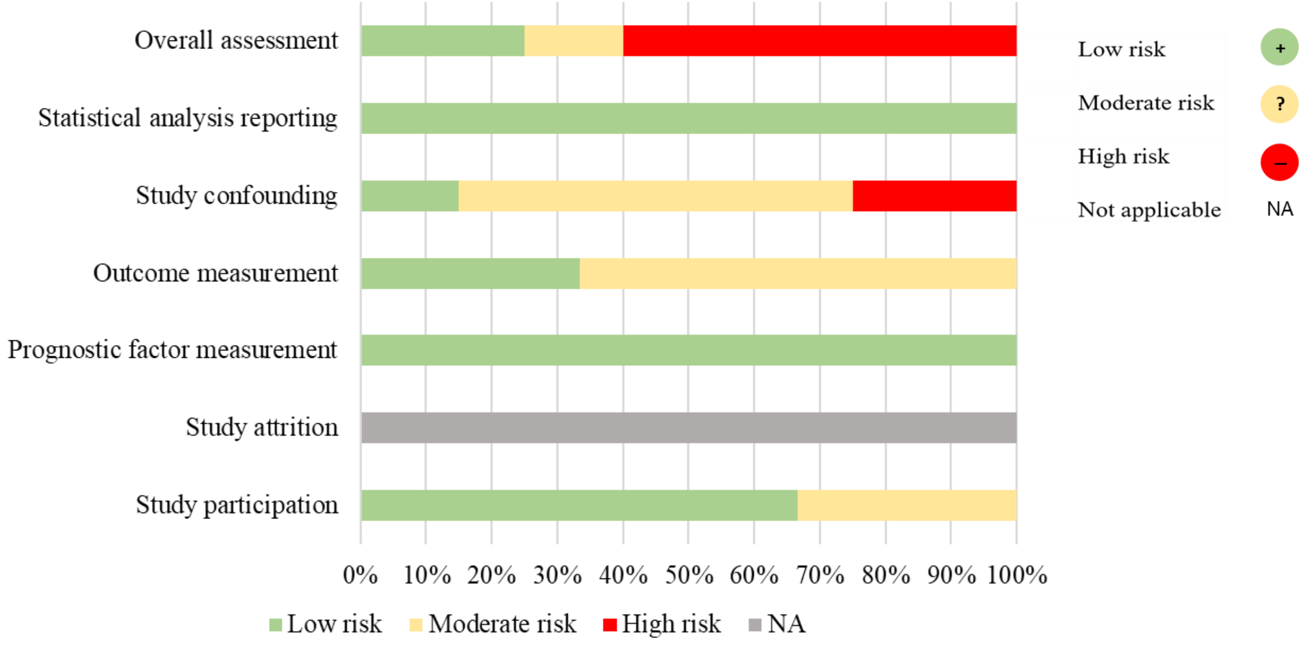

© 2022 Nagy R et al. *JAMA Network Open.*

eFigure 19. **Risk of bias assessment at study and domain level regarding fasting glucose.**

Study		Study participation	Study attrition	Prognostic factor measurement	Outcome measurement	Study confounding	Statistical analysis reporting	**Overall risk of bias**	**Included in the MA**
**Panagopoulou et al.,2013[10]**		3	NA	3	2	2	3	2	yes
**Bonhoure et al., 2020[3]**		3	NA	3	3	2	3	3	yes
**Jimenez et al., 2012[15]**		3	NA	3	3	2	3	3	yes
Total	low	3		3	2		3	2	
Total	moderate				1	3		1	
Total	high								
Total	NA		3						

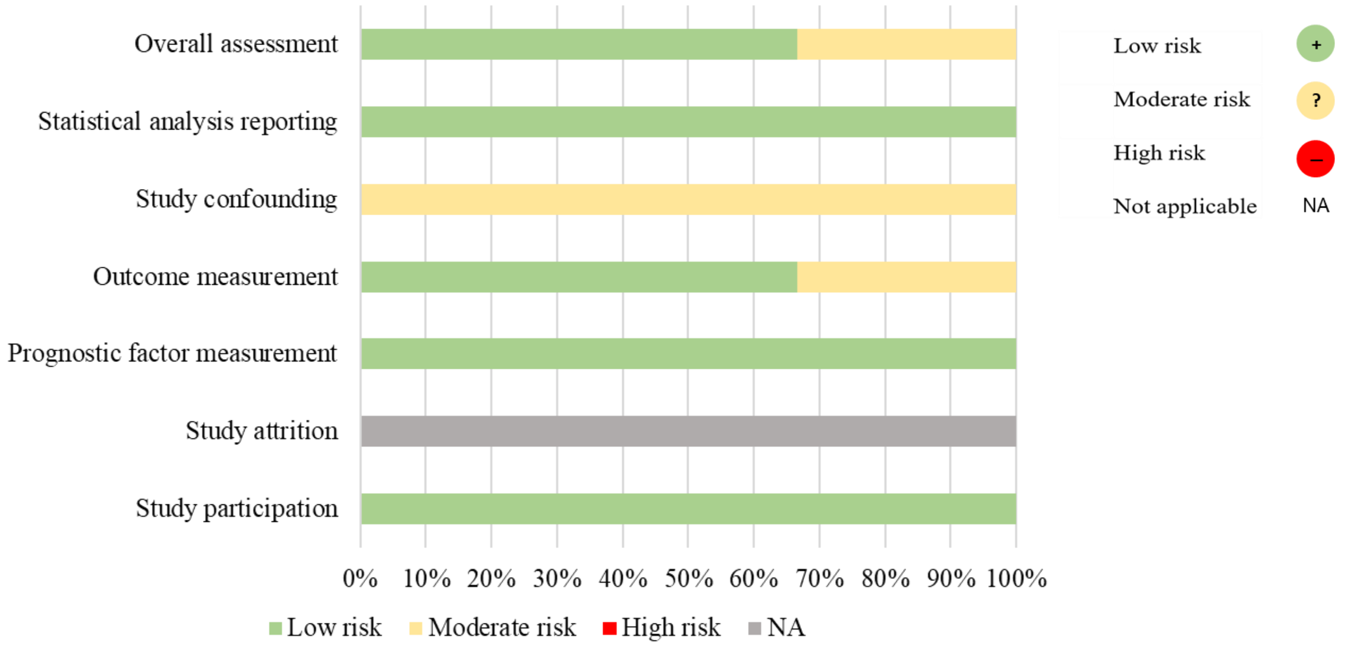

© 2022 Nagy R et al. *JAMA Network Open.*

eFigure 20. **Risk of bias assessment at study and domain level regarding HbA1c%.**

Study		Study participation	Study attrition	Prognostic factor measurement	Outcome measurement	Study confounding	Statistical analysis reporting	**Overall risk of bias**	**Included in the MA**
**Harindhanavudhi et al., 2020[4]**		2	NA	3	2	1	3	1	yes
**Bonhoure et al.,[3] 2020**		3	NA	3	3	2	3	3	yes
**Jimenez et al., 2012[15]**		3	NA	3	3	2	3	3	yes
Total	low	2		3	2		3	2	
Total	moderate	1			1	2			
Total	high					1		1	
Total	NA		3						

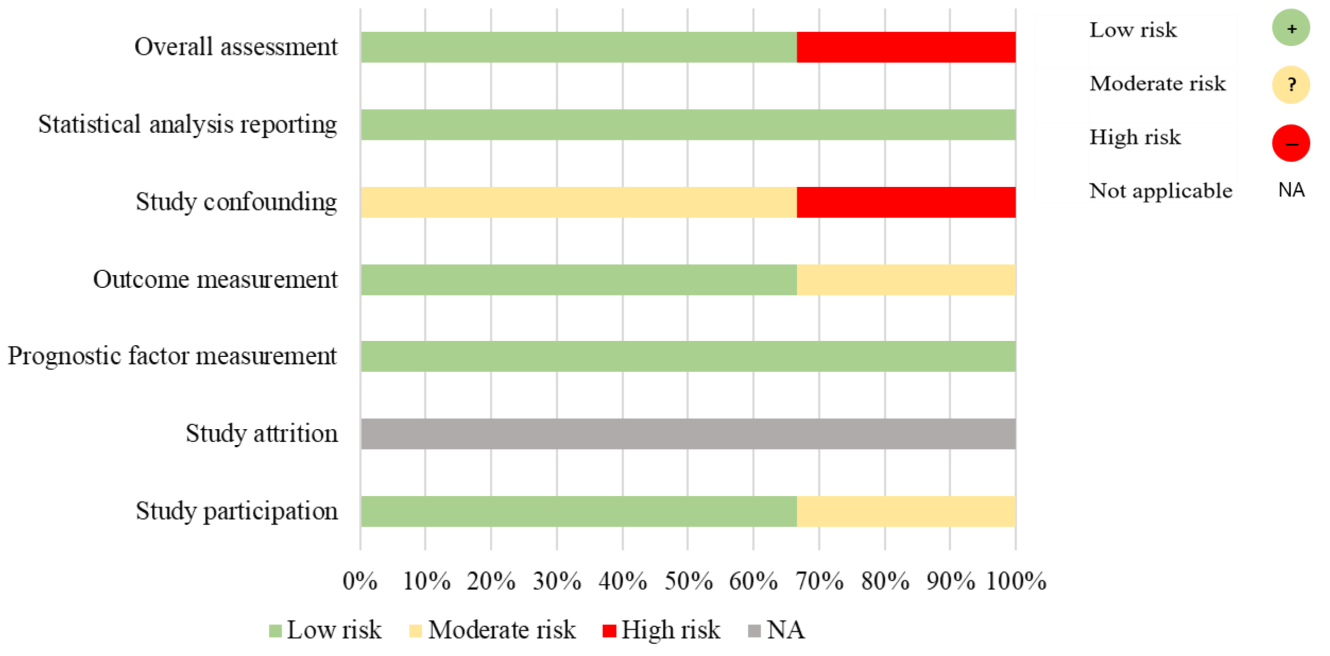

© 2022 Nagy R et al. *JAMA Network Open.*

eFigure 21. **Risk of bias assessment at study and domain level regarding total cholesterol.**

Study	Study participation	Study attrition	Prognostic factor measurement	Outcome measurement	Study confounding	Statistical analysis reporting	**Overall risk of bias**	**Included in the MA**
**Harindhanavudhi et al.,2020[4]**	2	NA	3	3	2	2	1	no
**Stephenson et al.,[2] 2013**	3	NA	3	3	1	3	1	yes
**Bonhoure et al., 2020[3]**	3	NA	3	3	2	3	3	yes
**Panagopoulou et al.,2013[10]**	3	NA	3	2	2	3	2	yes
**Jimenez et al., 2012[15]**	3	NA	3	2	2	3	2	yes
Total low	4					4	1	
Total moderate	1			4	4	1	2	
Total high			5	1	1		2	
Total NA		5						

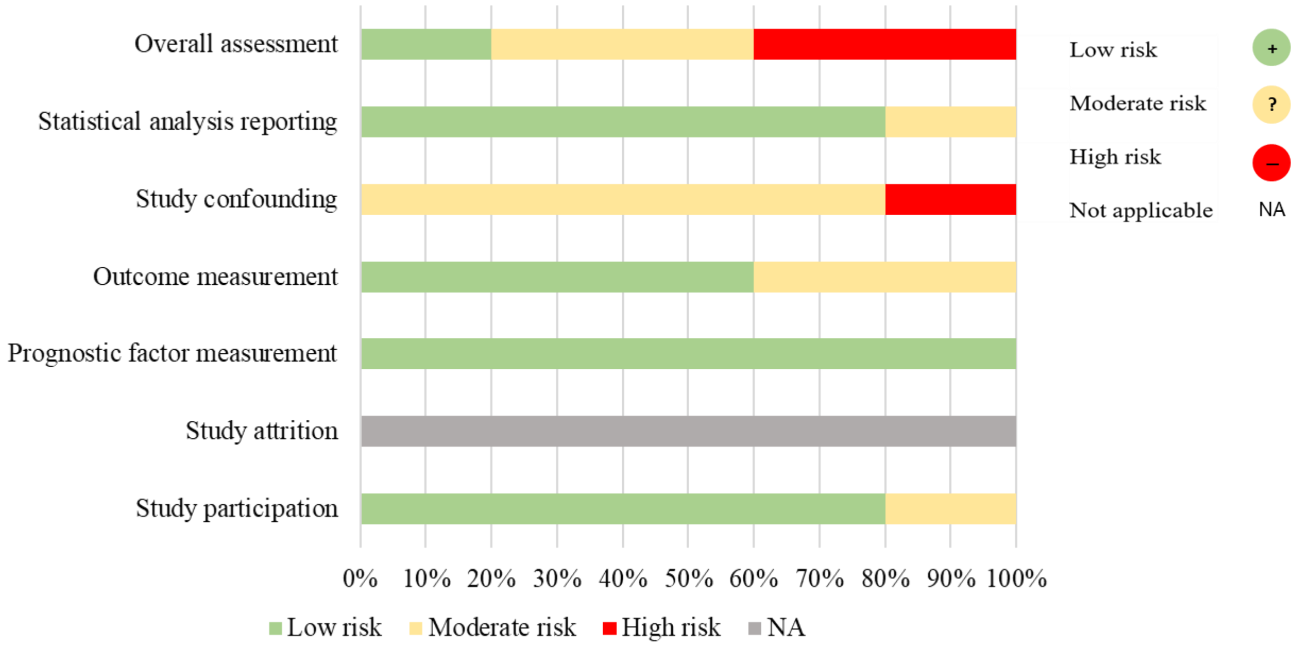

© 2022 Nagy R et al. *JAMA Network Open.*

eFigure 22. Risk of bias assessment at study and domain level regarding triglycerides

Study		Study participation	Study attrition	Prognostic factor measurement	Outcome measurement	Study confounding	Statistical analysis reporting	**Overall risk of bias**	**Included in the MA**
**Harindhanavudhi et al.,2020[4]**		2	NA	3	3	2	2	1	no
**Stephenson et al., 2013[2]**		3	NA	3	3	1	3	1	yes
**Bonhoure et al.,[3] 2020**		3	NA	3	3	2	3	3	yes
**Panagopoulou et al.,2013[10]**		3	NA	3	2	2	3	2	yes
**Jimenez et al., 2012[15]**		3	NA	3	2	2	3	2	yes
Total	low	4					4	1	
Total	moderate	1			4	4	1	2	
Total	high			5	1	1		2	
Total	NA		5						

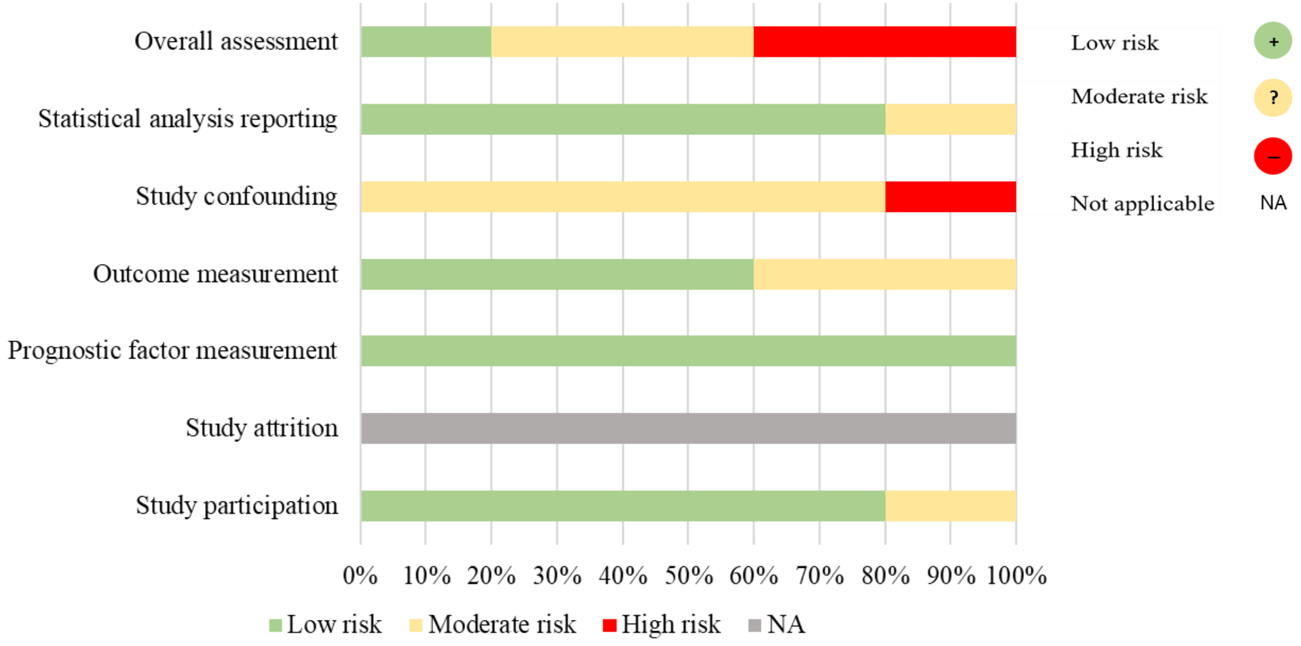

© 2022 Nagy R et al. *JAMA Network Open.*

eFigure 23. **Risk of bias assessment at study and domain level regarding *Pseudomonas aeruginosa* colonization**

Study		Study participation	Study attrition	Prognostic factor measurement	Outcome measurement	Study confounding	Statistical analysis reporting	**Overall risk of bias**	**Included in the MA**
**Stephenson et al., 2013[2]**		3	NA	3	3	1	3	1	yes
**Dray et al., 2004[13]**		2	NA	3	2	2	3	1	yes
**Ramirez et al., 2014[14]**		2	NA	3	2	2	3	1	yes
**Panagopoulou et al.,2013[10]**		3	NA	3	3	2	3	3	yes
**Barni et al.,2017[8]**		3	NA	3	3	3	3	3	yes
**Bodnar et al.,2014[9]**		3	NA	3	3	3	3	3	yes
Total	low	4		6	4	2	6	3	
Total	moderate	2			2	3			
Total	high					1		3	
Total	NA		6						

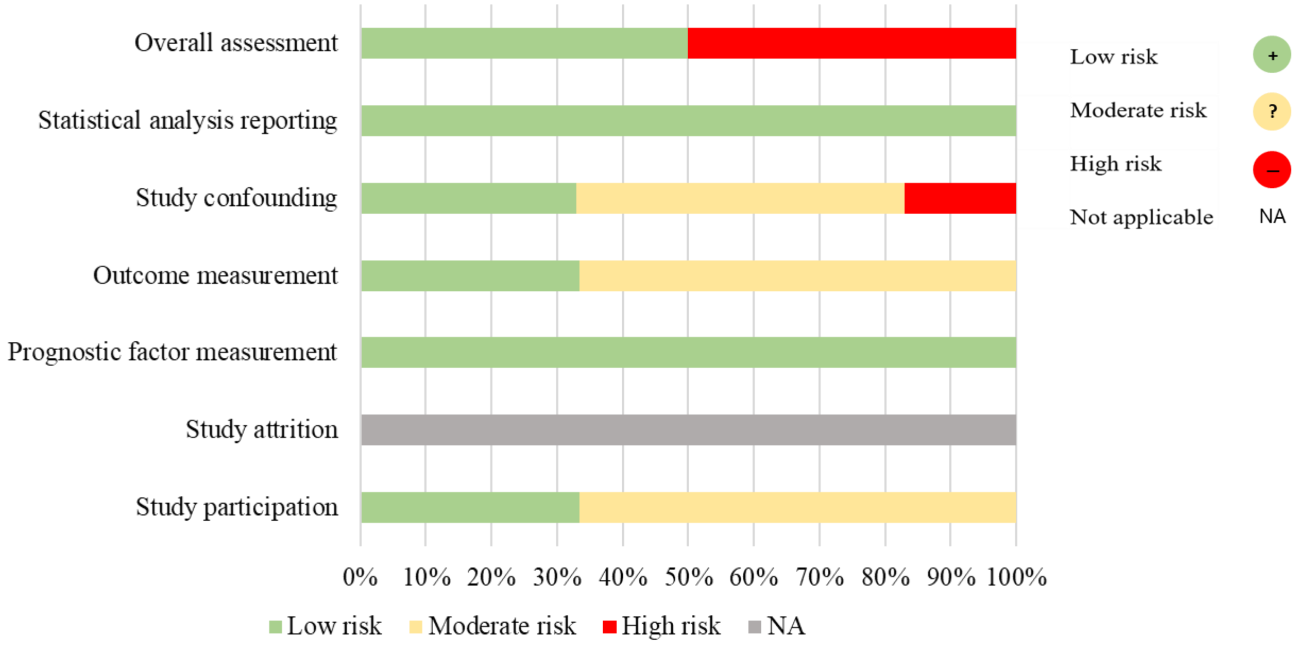

© 2022 Nagy R et al. *JAMA Network Open.*

eFigure 24. Risk of bias assessment at study and domain level regarding fasting insulin.

Study		Study participation	Study attrition	Prognostic factor measurement	Outcome measurement	Study confounding	Statistical analysis reporting	**Overall risk of bias**	**Included in the MA**
**Bonhoure et al., 2020[3]**		3	NA	3	3	2	3	3	yes
**Panagopoulou et al., 2013[10]**		3	NA	3	2	2	3	2	yes
**Jimenez et al., 2012[15]**		3	NA	3	3	2	3	3	yes
Total	low	3		3	2		3	2	
Total	moderate				1	3		1	
Total	high								
Total	NA		3						

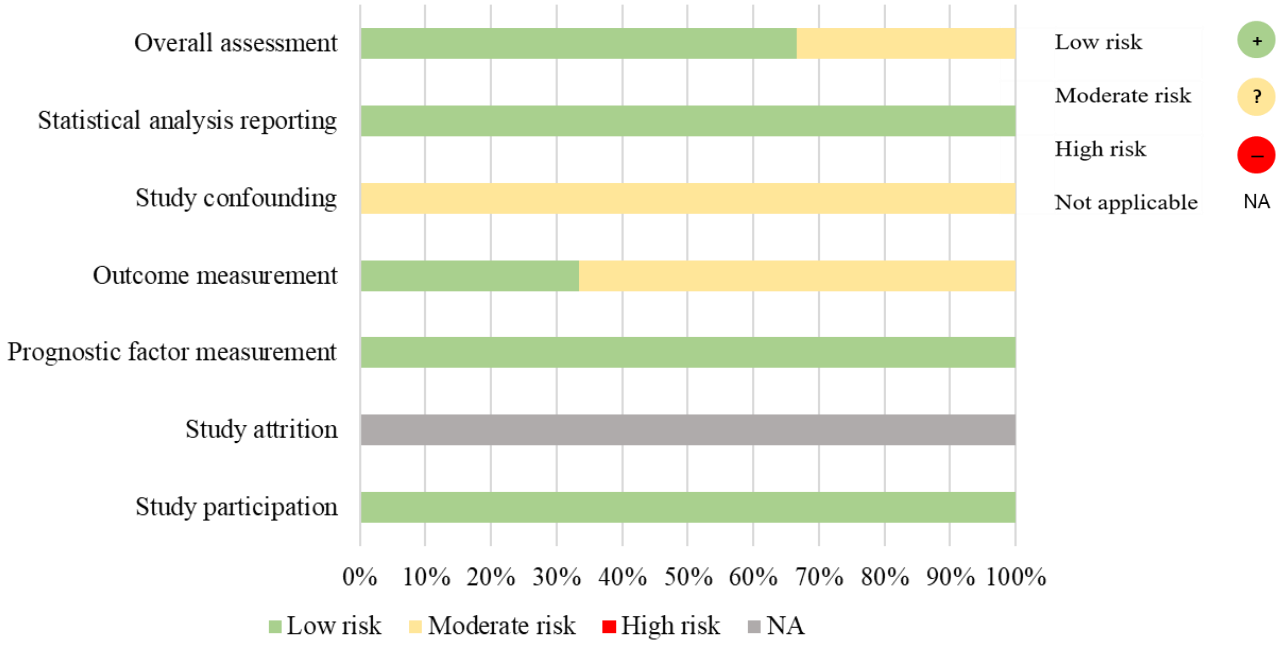

© 2022 Nagy R et al. *JAMA Network Open.*

eReferences

1. Gozdzik J, Cofta S, Piorunek T, Batura-Gabryel H, Kosicki J. Relationship between nutritional status and pulmonary function in adult cystic fibrosis patients. *Journal of Physiology and Pharmacology.* 2008;59(SUPPL. 6):253-260.
2. Stephenson AL, Mannik LA, Walsh S, et al. Longitudinal trends in nutritional status and the relation between lung function and BMI in cystic fibrosis: A population-based cohort study. *American Journal of Clinical Nutrition.* 2013;97(4):872-877.
3. Bonhoure A, Boudreau V, Litvin M, et al. Overweight, obesity and significant weight gain in adult patients with cystic fibrosis association with lung function and cardiometabolic risk factors. *Clinical Nutrition.* 2020;39(9):2910-2916.
4. Harindhanavudhi T, Wang Q, Dunitz J, Moran A, Moheet A. Prevalence and factors associated with overweight and obesity in adults with cystic fibrosis: A single-center analysis. *Journal of Cystic Fibrosis.* 2020;19(1):139-145.
5. Hanna RM, Weiner DJ. Overweight and obesity in patients with cystic fibrosis: A center-based analysis. *Pediatric pulmonology.* 2015;50(1):35-41.
6. Umławska W, Krzyzanowska M, Zielińska A, Sands D. Effect of selected factors associated with the clinical course of the disease on nutritional status in children with cystic fibrosis. *Advances in Clinical and Experimental Medicine.* 2014;23(5):775-783.
7. Engelen MPKJ, Schroder R, Van der Hoorn K, Deutz NEP, Com G. Use of body mass index percentile to identify fat-free mass depletion in children with cystic fibrosis. *Clinical Nutrition.* 2012;31(6):927-933.
8. Barni GC, Forte GC, Forgiarini LF, Abrahão CLO, Dalcin PTR. Factors associated with malnutrition in adolescent and adult patients with cystic fibrosis. *Jornal brasileiro de pneumologia : publicacao oficial da Sociedade Brasileira de Pneumologia e Tisilogia.* 2017;43(5):337-343.
9. Bodnar R, Kadar L, Holics K, et al. Factors influencing quality of life and disease severity in Hungarian children and young adults with cystic fibrosis. *Italian journal of pediatrics.* 2014;40(1).
10. Panagopoulou P, Fotoulaki M, Nikolaou A, Nousia-Arvanitakis S. Prevalence of malnutrition and obesity among cystic fibrosis patients. *Pediatrics International.* 2014;56(1):89-94.
11. González Jiménez D, Muñoz-Codoceo R, Garriga-García M, et al. Excess weight in patients with cystic fibrosis: is it always beneficial? *Nutricion hospitalaria.* 2017;34(3):578-583.
12. Cano Megías M, Guisado Vasco P, González Albarrán O, Lamas Ferreiro A, Máiz Carro L. Association of the relative change in weight and body mass index with lung function in teenagers and adults with cystic fibrosis: Influence of gender and diabetes. *Endocrinologia y Nutricion.* 2015;62(9):422-429.
13. Dray X, Kanaan R, Bienvenu T, et al. Malnutrition in adults with cystic fibrosis. *European journal of clinical nutrition.* 2005;59(1):152-154.
14. Ramírez I, Filbrun A, Hasan A, Kidwell KM, Nasr SZ. Improving nutritional status in a pediatric cystic fibrosis center. *Pediatric pulmonology.* 2015;50(6):544-551.
15. González Jiménez D, Bousoño García C, Rivas Crespo MF, et al. Insulin resistance in overweight cystic fibrosis paediatric patients. *Anales de Pediatria.* 2012;76(5):279-284.
16. Panagopoulou P, Fotoulaki M, Manolitsas A, Pavlitou-Tsiontsi E, Tsitouridis I, Nousia-Arvanitakis S. Adiponectin and body composition in cystic fibrosis. *Journal of Cystic Fibrosis.* 2008;7(3):244-251.
17. Kotsifas K, Antonogiannaki EM, Diamantea F, Inglezos I. Overweight and obesity in Greek adult cystic fibrosis patients. Description and comparison to patients with optimal BMI. *Journal of Cystic Fibrosis.* 2016;15:S103.

© 2022 Nagy R et al. *JAMA Network Open.*

18. Barja S, Espinoza T, Cerda J, Sánchez I. Growth and pulmonary function in Chilean children and adolescents with cystic fibrosis. *Revista medica de Chile.* 2011;139(8):977-984.
19. Pedreira CC, Robert RGD, Dalton V, et al. Association of body composition and lung function in children with cystic fibrosis. *Pediatric pulmonology.* 2005;39(3):276-280.
20. Asseiceira I, Mexia S, Martins T, Almeida Nunes P, Pereira L, Barreto C. Fat free mass deficit in children and adolescents with cystic fibrosis. What is the implication in pulmonary function? *Pediatric pulmonology.* 2017;52:S134.
21. Junge S, Stein L, Schlüter K, Hoyng C. Phase angle (PA) from bioelectrical impedance analysis (BIA) in children with cystic fibrosis (CF). *Journal of Cystic Fibrosis.* 2012;11:S121.
22. Mexia S, Asseiceira I, Rodrigues T, Pereira L, Barreto C. Nutritional intervention in paediatric cystic fibrosis patients leads to improvement of nutritional status-Preliminary results of a prospective study. *Journal of Cystic Fibrosis.* 2015;14:S114.
23. Cherniak A, Gorinova Y, Krasovsky S, et al. Lung function and its association with nutritional and functional status in patients with cystic fibrosis living in the Moscow region. *Journal of Cystic Fibrosis.* 2018;17:S92.
24. Vukic Dugac A, Ladic A, Tanackovic L, et al. Association of body composition and lung function in adult patients with cystic fibrosis - data from Croatia. *Journal of Cystic Fibrosis.* 2020;19:S47.
25. Bell SC, Bowerman AR, Davies CA, Campbell IA, Shale DJ, Elborn JS. Nutrition in adults with cystic fibrosis. *Clinical Nutrition.* 1998;17(5):211-215.
26. Calella P, Valerio G, Thomas M, et al. Association between body composition and pulmonary function in children and young people with cystic fibrosis. *Nutrition (Burbank, Los Angeles County, Calif).* 2018;48:73-76.
27. Coderre L, Fadainia C, Belson L, et al. LDL-cholesterol and insulin are independently associated with body mass index in adult cystic fibrosis patients. *Journal of Cystic Fibrosis.* 2012;11(5):393-397.
28. Er B, Çelebioğlu E, Yalçin E, et al. Factors associated with severe lung disease in an adult population with cystic fibrosis: A single-center experience. *Turkish journal of medical sciences.* 2020;50(4):945-952.
29. Ionescu AA, Evans WD, Pettit RJ, Nixon LS, Stone MD, Shale DJ. Hidden Depletion of Fat-Free Mass and Bone Mineral Density in Adults with Cystic Fibrosis. *Chest.* 2003;124(6):2220-2228.
30. Jarad NA, Higgs S, Jeffcote T, Giles K. Factors associated with reduced FEV1 in adult patients with cystic fibrosis in a relatively affluent area. *Chronic respiratory disease.* 2005;2(3):133-137.
31. Belson L, Fadainia C, Ziai S, Berthiaume Y, Coderre L, Rabsa-Lhoret R. Associations between pulmonary function improvement and body mass index in a large cohort of Cystic Fibrosis patients. *Canadian Journal of Diabetes.* 2011;35(2):214.
32. Cárdenas G, Segurola H, Garcia N, et al. Fat-soluble vitamins and lung function in adult patients with cystic fibrosis (CF). *Clinical Nutrition.* 2016;35:S83-S84.
33. Bianchi ML, Romano G, Saraifoger S, Costantini D, Limonta C, Colombo C. BMD and body composition in children and young patients affected by cystic fibrosis. *Journal of Bone and Mineral Research.* 2006;21(3):388-396.
34. Moço VJR, Lopes AJ, Vigário PS, et al. Pulmonary disease severity and peripheral muscle function as limiting factors for exercise capacity in adult patients with cystic fibrosis. *Isokinetics and Exercise Science.* 2013;21(3):219-226.
35. Spirevska L, Fustik S, Jakovska T, Zorcec T, Stamatova A. Malnutrition in adolescent and adult patients with cystic fibrosis. *Journal of Cystic Fibrosis.* 2018;17:S116.
36. Papalexopoulou N, Dassios TG, Lunt A, et al. Nutritional status and pulmonary outcome in children and young people with cystic fibrosis. *Respiratory medicine.* 2018;142:60-65.
37. Souza dos Santos Simon MI, Drehmer M, de Abreu E Silva FA, et al. Association of nutritional status, plasma, albumin levels and pulmonary function in cystic fibrosis. *Nutrición*

© 2022 Nagy R et al. *JAMA Network Open.*

hospitalaria : organo oficial de la Sociedad Española de Nutrición Parenteral y Enteral. 2011;26(6):1322-1327.
38. Bolton CE, Ionescu AA, Evans WD, Pettit RJ, Shale DJ. Altered tissue distribution in adults with cystic fibrosis. *Thorax.* 2003;58(10):885-889.
39. King SJ, Nyulasi IB, Strauss BJG, Kotsimbos T, Bailey M, Wilson JW. Fat-free mass depletion in cystic fibrosis: Associated with lung disease severity but poorly detected by body mass index. *Nutrition (Burbank, Los Angeles County, Calif).* 2010;26(7-8):753-759.
40. Sermet-Gaudelus I, Souberbielle JC, Ruiz JC, et al. Low bone mineral density in young children with cystic fibrosis. *American journal of respiratory and critical care medicine.* 2007;175(9):951-957.
41. Rochat T, Slosman DO, Pichard C, Belli DC. Body composition analysis by dual-energy x-ray absorptiometry in adults with cystic fibrosis. *Chest.* 1994;106(3):800-805.
42. Sripalan N, E. Moen I, L. Finstad P, K. Brekke H, Ekornes M, Gharagozlian S. NUTRITIONAL STATUS IN ADULTS WITH CYSTIC FIBROSIS AND PANCREATIC INSUFFICIENCY. *Clinical Nutrition.* 2019;38:S312.
43. Boat T, Hente E, Wang S, et al. Body composition of patients with CF at the time of pulmonary exacerbation. *Pediatric pulmonology.* 2019;54:254.
44. Sills D, Mitchell-Whyte M, Dewar J, Barr H. Normal weight obesity in cystic fibrosis is associated with glucose tolerance irregularities in cystic fibrosis at a UK adult cystic fibrosis centre. *Journal of Cystic Fibrosis.* 2020;19:S16.
45. Bellissimo MP, Ivie E, Tran P, et al. Body composition and bone health in adults with cystic fibrosis across the glucose tolerance spectrum. *Pediatric pulmonology.* 2017;52:464.

© 2022 Nagy R et al. *JAMA Network Open.*
